# Effects of water stress on secondary metabolism of *Panax ginseng* fresh roots

**DOI:** 10.1371/journal.pone.0312023

**Published:** 2024-11-27

**Authors:** Wei Zhang, Wenfei Liu, Liyang Wang, Pengcheng Yu, Xiaowen Song, Yao Yao, Xiubo Liu, Xiangcai Meng

**Affiliations:** 1 Department of Pharmacognosy, Heilongjiang University of Chinese Medicine, Harbin, Heilongjiang, China; 2 Jiamusi College, Heilongjiang University of Chinese Medicine, Jiamusi, Heilongjiang, China; Universidade de Coimbra, PORTUGAL

## Abstract

The roots and rhizomes of *Panax ginseng* C.A. Mey are commonly used herbal medicine in Asian countries. These components contain a large number of secondary metabolites known as ginsenosides, which serve as primary active ingredient. Environmental factors significantly influence the production of secondary metabolites, which are crucial for enhancing plant adaptability to ecological stress. *P*. *ginseng* is a shady plant that thrives in a constantly humid and temperate environment. However, it cannot withstand excessive moisture, making soil moisture a significant ecological stress affecting *P*. *ginseng* survival. In this study, we applied a water spray to maintain a water-saturated surface on 5-year-old fresh *P*. *ginseng* roots for a duration of 5 days, to establish a short-term water stress condition. The results revealed a notable increase in superoxide anion (O_2_^·-^), hydrogen peroxide (H_2_O_2_), and NADPH oxidase (NOX) activity (*p* < 0.01), as well as malondialdehyde (MDA) contents (*p* < 0.01) in both the main root and fibrous root of *P*. *ginseng*. Additionally, superoxide dismutase (SOD), catalase (CAT), peroxides (POD), ascorbate peroxidase (APX) and glutathione reductase (GR) activities also elevated significantly under water stress (*p* < 0.01). Ascorbic acid (AsA), glutathione (GSH) and oxidized glutathione (GSSG) contents also showed a marked increase (*p* < 0.01). The main root treated with water showed the most positive impact on the 5^th^ day. Water stress boosted the activities of key enzymes including 3-hydroxy-3-methylglutaryl coenzyme A reductase (HMGCR), farnesyl pyrophosphate synthase (FPS), squalene synthase (SS), squalene epoxidase (SE), and dammarenediol-II synthase (DS) involved in the ginsenoside biosynthesis pathway (*p* <0.01). This resulted in a significant an increase in the level of ginsenosides Rg_1_, Rb_1_, Rf, Rg_2_+Rh_1_, Rc, and Rb_3_, by 42.4%, 21.0%, 15.7%, 157.9%, 18.3%, and 10.6% respectively, and an increase of 40.1% in total saponins content. Similarly, the fibrous root changes in the treated sample showed the most positive impact on the 4^th^ day. Specifically, Rg_1_, Re, Rb_1_, Rf, Rg_2_+Rh_1_, Rc, Ro, and Rb_2_ increased by 41.8%, 20.5%, 17.3%, 84.3%, 30.7%, 35.6%, 8.6%, and 7.6%, respectively, and an increase of 4.2% in total saponins content. Furthermore, 1,3-disphosphoglycerate (1,3-DPG) contents and phosphoenolpyruvate carboxylase (PEPC) activities, which are key intermediate of primary metabolism, were significantly elevated under water stress (*p* < 0.01). This indicates that the primary source of the raw materials used in the biosynthesis of secondary metabolites is sugars. Pharmacodynamic analysis demonstrated that water stress could increase the contents of ginsenosides, improve the quality of ginseng, and enhance the efficacy of ginseng root to a certain extent.

## Introduction

Plants cannot move to escape harsh ecological stress such as high temperatures, drought, floods, and other adversities. Under ecological stress, increased levels of abscisic acid levels cause the closure of stomata, leading to a rise in intracellular O_2_ produced by photosynthesis. Meanwhile, the photosynthetic electron transport system experiences excessive reduction, resulting in an improved conversion of oxygen into O_2_^·-^. Subsequently, the O_2_^·-^ is further converted into H_2_O_2_ and other products (Mehler reaction) [1,2]. The over-production of Reactive Oxygen Species (ROS) can cause damage to molecular structures, bio-membrane, DNA and peptide chain, and proteins, ultimately leading to metabolic dysregulation or cell death [3,4]. The antioxidant enzymes, such as SOD, CAT, POD, APX, etc., are the primary substances that against ROS. However, these antioxidant enzymes are also proteins that depend on hydrogen bonds and disulfide bonds to preserve their secondary and tertiary structures, ensuring their optimal functionality. Elevated levels of ROS can disrupt these bonds, causing the enzymes to lose their effectiveness. Plants can survive under severe ecological stress due to the development of a sophisticated secondary metabolism that helps eliminate or neutralize excessive ROS [5]. Secondary metabolites, including polyphenols (flavonoids), saponins, and alkaloids, etc, play critical roles in multiple ecological processes [6–8]. Once environmental stress occurs, plants respond by inducing the production of secondary metabolites to counteract ROS. Therefore, the levels of ROS can be regulated by antioxidant enzymes, secondary metabolites, and other antioxidant systems, ultimately maintained at a stable level [9].

Ginseng (*Panax*. *ginseng* C. A. Meyer) is a perennial herb derived from the genus Panax in the family Araliaceae. Its medicinal parts are roots and rhizomes, which are called "the king of herbs". They possess various effects such as anti-aging, regulation of the nervous system, improvement of blood circulation, and regulation of the endocrine system, etc. [10–12].

Flavonoids have a strong ability to eliminate ROS as a result of their higher concentration of -OH and -OCH_3_ groups [13–15]. However, ginseng’s primary active components are secondary metabolite saponins, including Rg_1_, Re, Rg_2_, Rb_1_, Rc, and Rb_2_, etc. [16]. Consequently, because flavonoids are sparsely present in ginseng, they demonstrate a relatively limited ability to adapt to diverse environments [17,18]. The biosynthesis of these ginsenosides proceeds as follows: acetyl-CoA generating isopentenyl pyrophosphate (IPP) and dimethylallyl diphosphate (DMAPP) through the mevalonate (MVA) or methylerythritol phosphate (MEP) pathways. This is followed by the catalysis of 2,3-oxidosqualene by enzymes such as FPS, SS, and SE, resulting in the formation of various ginsenoside monomers through a sequence of cyclization, hydroxylation, and ring functional group glycosylation processes [19]. It has been reported that soil moisture is the main stress for ginseng. Both excessive drought and excessive humidity cause root damage [20]. *P*. ginseng can only survive in an environment with stable temperature and humidity. The growth of ginseng and the synthesis of ginsenosides are enhanced when the relative soil water content ranges from 60% to 80% [21]. Additionally, a field water holding capacity of around 30% ± 5% boosts the total ginsenosides content as well as ginsenosides Rg_1_ and Re. On the other hand, a temporary field capacity of 90% ± 5% increases the ginsenoside Rb_1_ content [22], suggesting that water stress leads to an increase in ginsenoside content.

The secondary metabolism of plants is closely related to the environment. Appropriate stress can enhance plant secondary metabolism, which usually are medicinal components. Consequently, short-term ecological stress can increase the quality of the medicinal materials. The post-harvest ginseng fresh root is a living organ with a complete metabolic system. This study focuses on exploring the relationship among ROS, antioxidant system, and secondary metabolites of ginseng under excessive water conditions. The fresh root of ginseng is used as the subject of study in vitro, to uncover the biological mechanism of water stress on ginsenosides. This research provides both a theoretical foundation and practical techniques to produce high-quality ginseng.

## Materials and methods

### Plant materials

Fresh 5-year-old roots of cultivated ginseng, identified by Prof. Xiang-cai Meng of Heilongjiang University of Chinese medicine, were collected in Ji’an County, Jilin Province, China, collected in May 2023 and wrapped in moss to maintain freshness.

### Instrument

CP225D Electronic balance (Shanghai Precision Instrument Co., Ltd.), SD40 Ice making Machine (Guangzhou Guangkun Electric Appliance Manufacturing Co., Ltd.), 752 UV-vis Spectrophotometer (Shanghai Jinghua Science and Technology Instrument Co., Ltd.), 98-1-B Electronic temperature regulating Electric heating sleeve (Tianjin Tester instrument Co., Ltd.), TGL- 16LM desktop high-speed freezing centrifuge (Hunan Star Science Instrument Co., Ltd.), JulaboTW20 digital display constant temperature water bath pot (Julabo Co., Ltd., Germany). QL-902 Vortex oscillator (Haimen Qilin Bell instrument Manufacturing Co., Ltd.); Thermo enzyme labeling instrument (Thermo Co., Ltd.); DHG- 9015A blast drying oven (Shanghai-Heng Scientific instrument Co., Ltd.); Shimadzu High performance liquid Chromatography (LC2010A).

### Reagent

O_2_^·-^ free radical determination kit (NO.2309002), protein quantification (TP) kit (20230420), H_2_O_2_ kit (20230726), MDA kit (20230422). SOD kit (20230624), CAT kit (20230503), POD kit (20230616). 1,3-DPG (202307), PEPC kit (202306), NOX kit (202303), APX kit (20230521), AsA kit (NO.1012304011), GR kit (NO.1012304011), GSH determination kit (NO.1012303305), GSSH determination kit (NO.1012304041), HMGCR kit (202303), FPS kit (202303), SS kit (202303), SE kit (202303), DS kit (202303), HPLC grade acetonitrile (R142190), phosphoric acid chromatograph pure (TEDIA Co., Ltd.); The ginsenoside standards, Rg_1_, Re and Rb_1_ were purchased from the National Institute for the Control of Pharmaceutical and Biological Products (Beijing, China), ginsenoside standards, Rf, Rg_2_, Rh_1_, Rc, Ro, Rb_2_, Rb_3_, were purchased from Shanghai Yuanye Biotechnology Co., Ltd. (Shanghai, China), the purity is more than 98%. D101 macroporous adsorption resin (Tianjin Huida Chemical Co., Ltd.), vanillin (Tianjin Guangfu Fine Chemical Research Institute), perchloric acid (Tianjin Fuyu Fine Chemical Co., Ltd.), glacial acetic acid (Tianjin Tianli Chemical Reagent Co., Ltd.).

### Experimental method

Selected fresh ginseng roots with a similar diameter to main root was divided into two groups. The treatment group sprayed water uniformly to the surface until saturated, replenished water once a day for 5 days. In contrast, the control group was covered with moss to maintain its freshness. The samples of two groups of ginsengs were placed in an artificial intelligence incubator at 15°C/10°C. Root samples were collected at 0, 1, 2, 3, 4 and 5 d of treatment, with several roots (n ≥ 6) being collected simultaneously. First, the main root phloem and fibrous roots were cut into separate pieces of 0.30 g × 30 samples per tissue type. The samples were used to evaluate the activities of various enzymes, including SOD, CAT, POD, NOX, PEPC, HMGCR, FPS, SS, SE, DS, and the content of 1,3-DPG. Additionally, 0.1 g × 30 samples of each piece were taken for the determination of APX and GR activities, as well as the content of O_2_^·-^, H_2_O_2_, MDA, AsA, GSH, and GSSG. All samples were sealed with aluminum foil and stored at -80°C. Next, more than 30 g of the main roots and fibrous roots were dried in an oven at 55°C, then crushed by an ultra-fine grinder and sifted through a 65-mesh sieve for the determination of Rg_1_, Re, Rf, Rg_2_+Rh_1_, Rb_1_, Rc, Ro, Rb_2_ and Rb_3_. Each index measurement was repeated three times.

#### Determination of related indexes of ROS metabolism and antioxidant system

*Determination of ROS in fresh roots of ginseng*. The content of homogenate protein in fresh root of *P*. *ginseng* was determined with the commercially available TP assay kit (Nanjing Jiancheng Bioengineering, Nanjing, China) and expressed in g/L. The O_2_^·-^and H_2_O_2_ contents were measured using a commercially available assay kit (Beijing Solarbio Science & Technology, Beijing, China and Nanjing Jiancheng Bioengineering, Nanjing, China), expressed in μmol/g and mmol/g, respectively.

*Determination of MDA content*. The MDA contents were measured using a commercially available assay kit (Nanjing Jiancheng Bioengineering, Nanjing, China) and expressed in nmol/g.

*Determination of ROS-related enzyme activity*. The activity of NOX was determined with a commercially available assay kit (Jiangsu jingmei Science & Technology, Yancheng, China) and expressed in ng/L. Evaluation of the SOD, CAT, and POD activities was expressed as U/g FW according to the protocol of a commercial assay kit (Jiancheng Bioengineering, Nanjing, China).

*Determination of parameters related to AsA-GSH cycle*. The AsA, GSH, and GSSG contents were measured using a commercially available assay kit (Beijing Boxbio, Beijing, China), expressed as μmol/g, μg/g, and μg/g, respectively. Evaluation of APX and GR activities were expressed as U/g FW according to the protocol of a commercial assay kit (Jiancheng Bioengineering, Nanjing, China).

#### Determination of secondary metabolism-related enzyme activity

The activities of HMGCR, FPS, SS, SE, and DS were determined with a commercially available assay kit (Jiangsu jingmei Science & Technology, Yancheng, China) and expressed in ng/L.

#### Determination of the ginsenosides contents in the fresh roots of *P*. *ginseng*

*Monomer ginsenosides*. The contents of ginsenosides Rg_1_, Re, Rf, Rg_2_, Rh_1_, Rb_1_, Rc, Ro, Rb_2,_ and Rb_3_ were determined by HPLC, following the method described by Lin *et al*. [23]. 1.0 g of both the main root and fibrous root powder of ginseng were precisely measured and placed in a 50 ml cork triangle container. The extraction process involved using methanol and ultrasonic at 100 Hz for 40 min and filtered by 0.22 μm filter for HPLC analysis. Each sample was repeated three times. The ginsenoside standards of Rg_1_, Re, Rf, Rg_2_, Rh_1_, Rb_1_, Rc, Ro, Rb_2_, and Rb_3_ were weighed precisely, then placed in a 5 ml centrifuge tube, dissolved with methanol, and adjusted volume to prepare a ginsenoside mixed standard solution for use.

The prepared samples were analyzed using an Agilent 1260 HPLC system (Agilent, United States) and separated in a C_18_ column (250 mm × 4.6 mm, ID 5 μm). The column temperature was 30°C, the flow rate was 1.0 mL/min, the injection volume was 10 μL, and UV measurements were obtained at 203 nm. The mobile phase was composed of A (0.1% phosphoric acid water) and B (acetonitrile), the gradient elution was performed as follows: 0~20 min, 18%~20% B; 20~60 min, 20%~24% B; 60~80 min, 24%~30% B; 80~110 min, 30%~34% B.

*Total ginsenosides*. The total ginsenosides were determined by using the vanillin-glacial acetic acid-perchloric acid colorimetric method reported by Kubo *et al* [24]. And ginsenoside Re was employed as standard. The absorbance (Y) was ordinate and the solution mass (X) was Abscissa. The standard curve was drawn to calculate the total ginsenosides content.

*Method validation for quantitation*. The linear relationship of 10 active components, the precision of the instrument, the stability of the solution within 24 h, the repeatability of the method, and the recovery rate were investigated respectively.

#### Determination of the intermediate metabolite contents of glycolysis PEPC activity

1,3-DPG contents and PEPC activities were determined with a commercially available assay kit (Jiangsu jingmei Science & Technology, Yancheng, China) and expressed in nmol/L and ng/L respectively.

#### Pharmacodynamic verification

*Drug preparation*. The method described by Song *et al* was referenced [25]. The 0^th^ day ginseng main root and the ginseng main root on the 5^th^ day with water stress were dried to constant weight and crushed into coarse powder. An appropriate amount of ginseng was taken, and 10 times the amount of water was added. The mixture was soaked for 12 h, then boiled for 1 h, and filtered, the extraction process was repeated three times. The filtrates were then combined and concentrated to achieve 0.039 g/ ml crude drug for further use.

*Animal experiments*. The 8-week-old male Kunming mice (weighing 18~24 g), provided by Liaoning Changsheng Biotechnology Co., Ltd with Certificate of Quality No. SCXK (Liao)-2020-0001 (Benxi, China), were raised in a ventilated and dry environment, with a temperature of 20~23°C, a relative humidity of 50~60%, and a 12 h light/dark cycle. They had free access to food and water. All animals’ treatments and experiments were carried out after being approved by the Animals Laboratory Ethical Committee of Heilongjiang University of Chinese Medicine (Approval Number: SYXK2023-122913).

After suitable feeding for 7 days, the mice were randomly divided into 6 groups (n = 10): the blank control group (administrated sterile saline and injected with saline); the model group (D-galactose (120 mg/kg/d) was subcutaneously injected with a volume of 0.01 ml/g, qd×42); the positive control group (from the 16^th^ day of the establishment of D-galactose model, the mice were administrated with donepezil hydrochloride (1.3 mg/kg/d) qd×27); the ginseng main root group on the 0^th^ day (from the 16th day of the establishment of D-galactose model, the mice were administrated with 0-day ginseng decoction (0.39 g/kg/d) qd×27); the ginseng main root group with water stress on the 5^th^ day (from the 16th day of the establishment of D-galactose model, the mice were administrated with the 5^th^ ginseng decoction (0.39 g/kg/d) qd×27).

*Analysis of Morris water maze*. The evaluation of the spatial learning and memory abilities of mice referred to the method described by Li *et al* [26] with minor modifications. In detail, training mice to find a circular platform (10 cm in diameter, 1 cm underwater) located in the middle of any quadrant in 2 min. After the beginning of the experiment, the instrument automatically recorded the time and trajectory of the mice till finding the platform, the time of the platform being found was the incubation period. All the indicators are averaged.

*Histopathological staining analysis*. After the water maze experiment, mice were anesthetized by intraperitoneal injection of urethane (0.008 ml/g) as reported by Yu *et al* [27], the brain tissue was perfused with 4% paraformaldehyde and fixed for 24 h, rinsed with the slow flow for 12 h, dehydration with gradient ethanol and xylene, then soaked in melted wax and embedded, sliced to 5~8 μm. The sections were stained with HE reagent and the pathological changes in the hippocampus of mice were observed by light microscope (Nikon, Eclipse Ci-L, Japan).

*Analysis of oxidative stress indexes in brain tissues*. The method described by Zhang *et al*. was referenced [28]. The mice brain tissues of appropriate size in each group were rinsed quickly in precooled normal saline, and the residual fluid on the surface of the brain tissue was discarded with filter paper. After preparing the 10% brain tissue homogenate, the supernatant was collected at 3500 rpm centrifugation for 10 min. SOD, glutathione peroxidase (GSH-PX) activities and MDA contents of the brain tissue were determined using commercial kits (Jiancheng Bioengineering, Nanjing, China), expressed in U/mg, U/mg, and nmol/mg, respectively.

### Statistical analysis

Microsoft Office Excel 2007 (Microsoft Corp., Redmond, WA, United States) was used to sort the original data. Origin 2021 (OriginLab, Northampton, MA, United States) was used for graphic illustration. The whole data was expressed in mean ± standard deviation (Mean ± S.D.) and IBM SPSS 27.0 (IBM Corp., Armonk, NY, United States) was used for the independent samples *t*-test. The statistically significant differences were thought to be *p < 0.05 or **p < 0.01.

## Results

### Effect of water stress on ROS

#### O_2_^·-^ and H_2_O_2_ contents

Compared with the 0 d, the contents of O_2_^·-^ and H_2_O_2_ in the control group remained essentially unchanged at all times. However, in the water stress group, the levels of O_2_^·-^ and H_2_O_2_ increased to various degrees during the stress period, indicating that excessive water has produced stress to ginseng.

The contents of O_2_^·-^ in the main root exhibited a pattern of decrease-increase-decrease. It peaked on the 3^rd^ day of stress, showing a 14.4% increase over the 0^th^ day. The content of H_2_O_2_ firstly decreased and then increased, and peaked on the 5^th^ day, with a 28.3% enhancement compared to that on the 0^th^ day.

**Fig 1:**
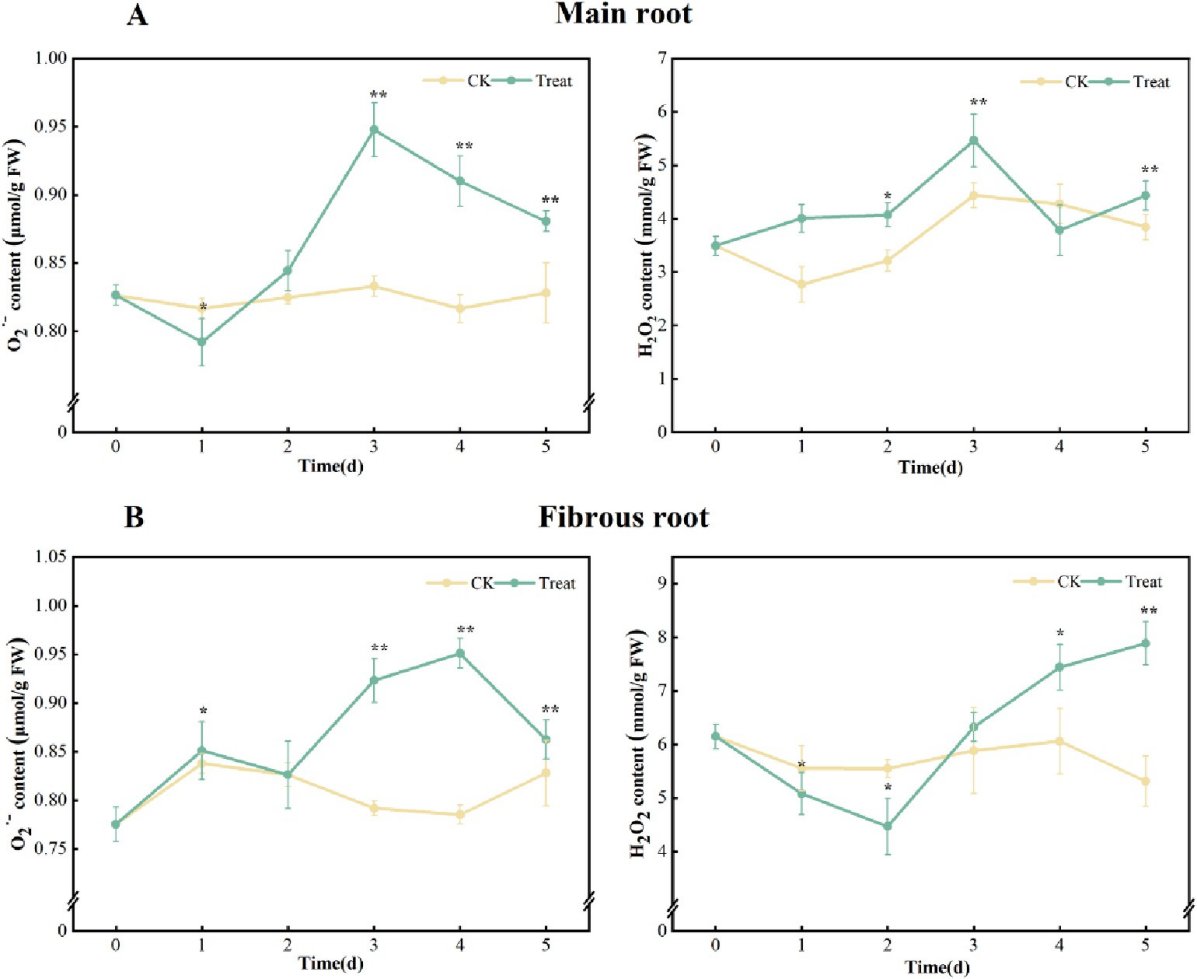
O2·- and H_2_O_2_ contents in *P*. *ginseng*. (A) main root, (B) fibrous root.

The contents of O_2_^·-^ in the fibrous root of stress increased, and peaked on the 4^th^ day, with a 21.8% higher level than that on the 0^th^ day. H_2_O_2_ exhibited an increase-decrease course, and peaked on the 3^rd^ day, with a 56.7% higher level than that on the 0^th^ day (Fig 1).

#### NOX activity

Compared with the 0 d, the NOX activities in the main root of the control group had a little change, but they increased slightly in the fibrous root, reaching their highest on the 3^rd^ day with a growth of 33.0% compared to the control group. The concentration of NOX in the water stress group increased notably during the duration of stress.

**Fig 2:**
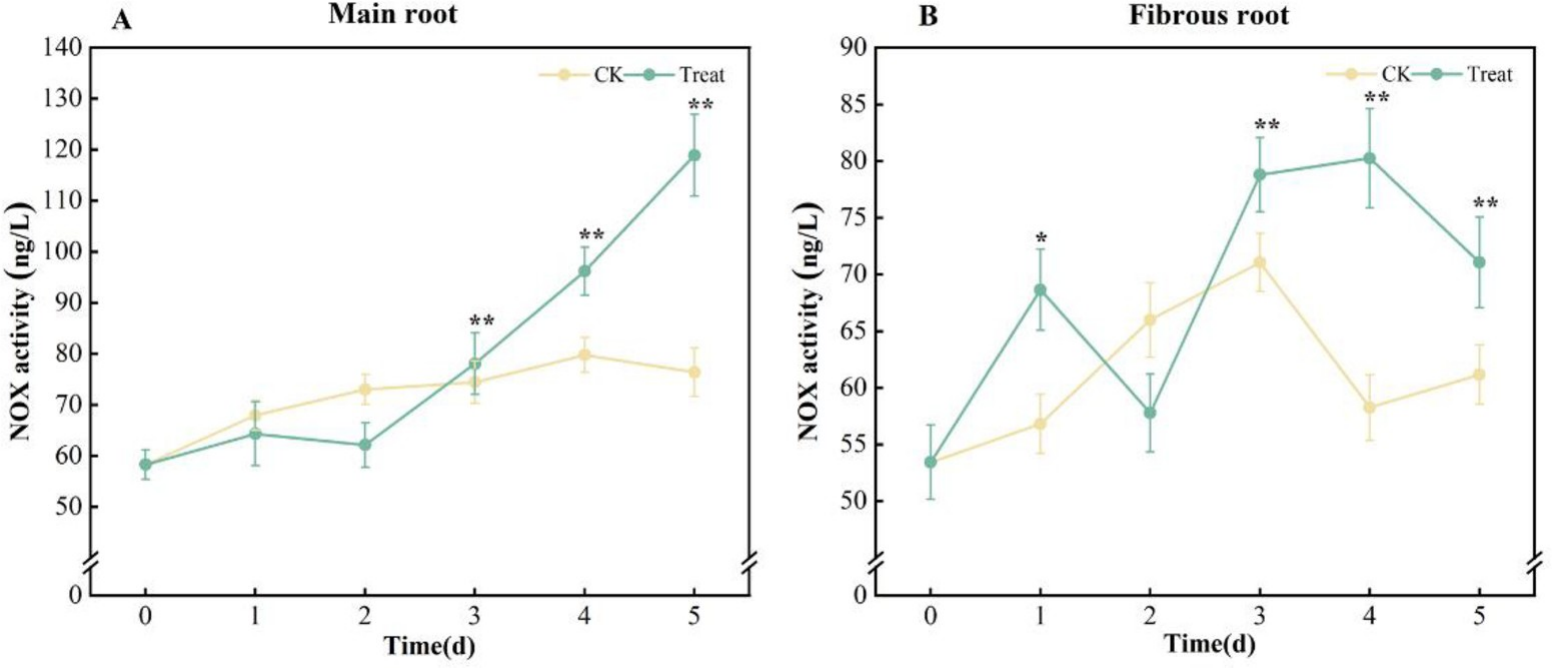
NOX activities in *P*. *ginseng*. (A) main root, (B) fibrous root.

The activities of NOX in the ginseng main root showed a linear increase from 2 to 5 days, peaking on the 5^th^ day, with a 104.1% elevation from that on the 0^th^ day. The fibrous roots showed a gradual growth, reaching their highest on the 4^th^ day with a 50.2% rise compared to the 0^th^ day (Fig 2).

#### MDA content

Compared with the 0 d, the contents of MDA in the root of the control group remained relatively stable over time. However, in the late stages, there was a little increase in the levels of MDA in the fibrous root, reaching a level that was 46.1% higher than that in the control group on the 4^th^ day.

**Fig 3:**
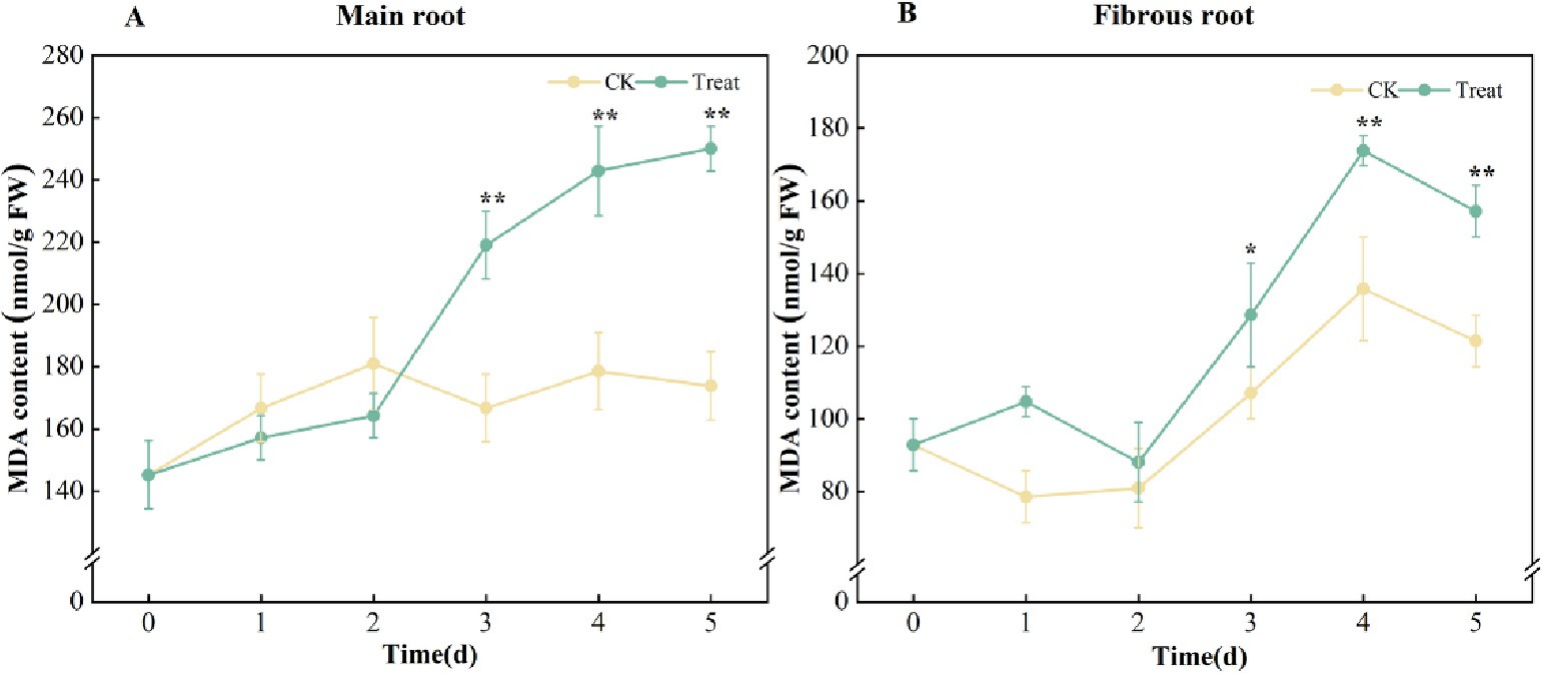
MDA contents in *P*. *ginseng*. (A) main root, (B) fibrous root.

The MDA contents in the main roots showed a modest increase in the initial phase of water stress. However, it increased significantly after the 2^nd^ day (*p* < 0.01) and peaked on the 5^th^ day, with a 72.2% increase compared with the 0^th^ day. The MDA contents in fibrous root first increased, and showed a maximum significant difference on the 4^th^ day (*p* < 0.01), with an 87.1% higher value than that on the 0^th^ day (Fig 3). These indicated that water stress did damage to ginseng.

#### Antioxidant enzyme activity

Compared with the 0 days, the activities of SOD, CAT, and POD in the control group remained constant over time. However, in the water stress group, the activities of all three antioxidant enzymes in both the ginseng main root and fibrous root increased.

The SOD activities in the ginseng main root exhibited a trend of decrease-increase-decrease during stress, peaking on the 4^th^ day. The CAT activities increased, peaking on the 4^th^ day. The POD activities were enhanced continuously and peaked on the 5^th^ day. Compared with the 0 days, the activities of SOD, CAT, and POD in ginseng main root grew by 14.5%, 39.8% and 19.3%, respectively.

**Fig 4:**
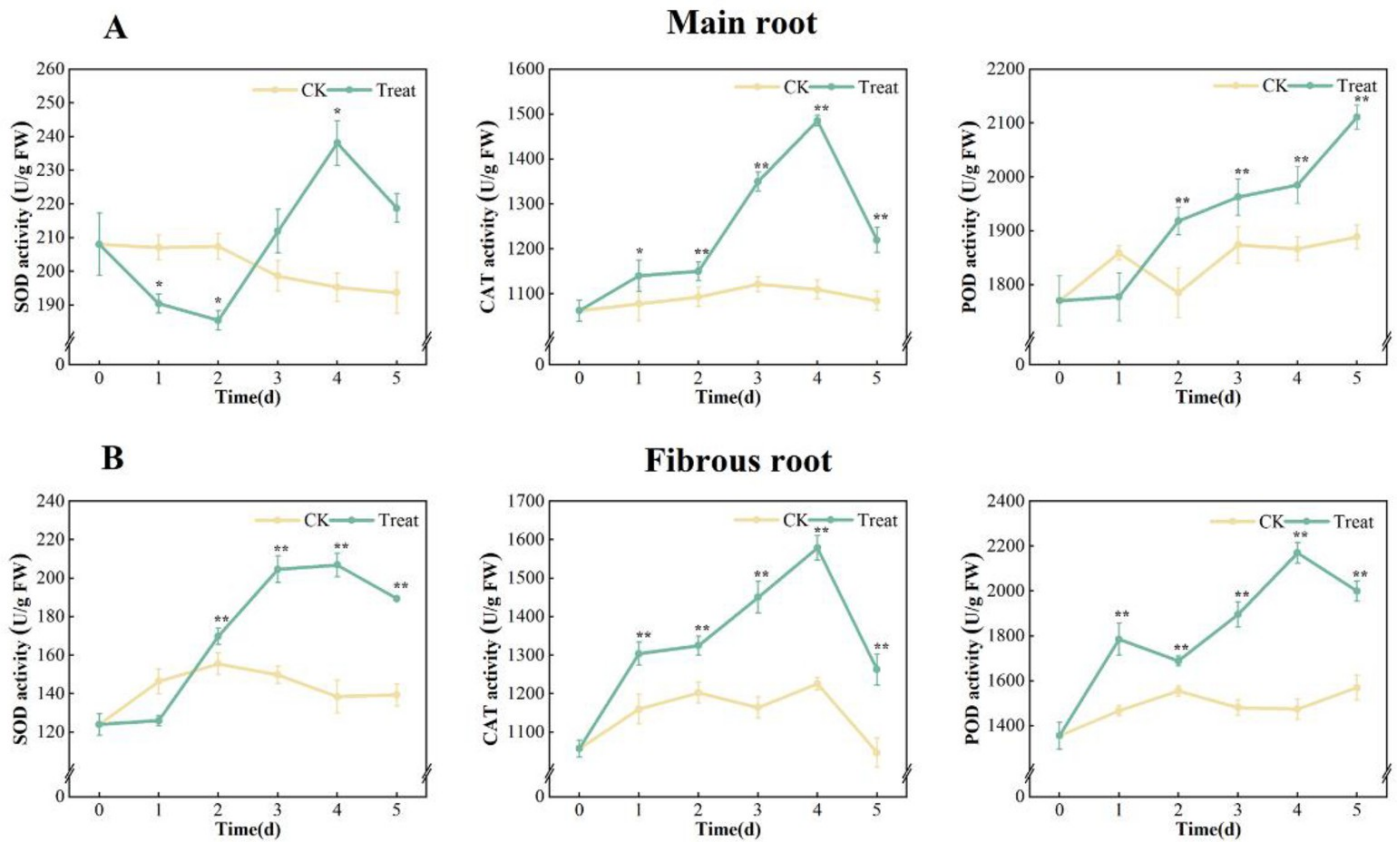
SOD, CAT and POD activities in *P*. *ginseng*. (A) main root, (B) fibrous root.

The activities of SOD in stressed fibrous root increased significantly on the first day (*p* < 0.01), peaking on the 3^rd^ day, and subsequently remained rather stable. Overall, the activities of CAT and POD showed an increase-decrease trend, and both peaked on the 4^th^ day. The activities of three antioxidant enzymes in fibrous root were increased by 66.9%, 49.3% and 60.1% respectively compared to 0 days (Fig 4).

#### AsA-GSH cycle

Compared with the 0 days, the control group showed minimal change in the AsA-GSH cycle, but water stress caused distinct modifications in the parameters of the AsA-GSH cycle in the ginseng main root and fibrous root.

During the stress, the AsA contents and the APX activities exhibited a decrease-increase-decrease trend, peaking on the 3^rd^ day, with an increase of 55.5% and 51.7% compared with the 0 days, respectively. The contents of GSH increased gradually and peaked on the 5^th^ day, increasing by 32.4% compared with the 0 days. The contents of GSSG and the activities of GR decreased at first and then increased, peaking on the 5^th^ day, with a rise of 37.7% and 58.1%, respectively, compared with the 0 days. The ratio of GSH/GSSG increased at first and then decreased, peaking on the 2^nd^ day, with a 37.1% higher than that on the 0 days.

**Fig 5:**
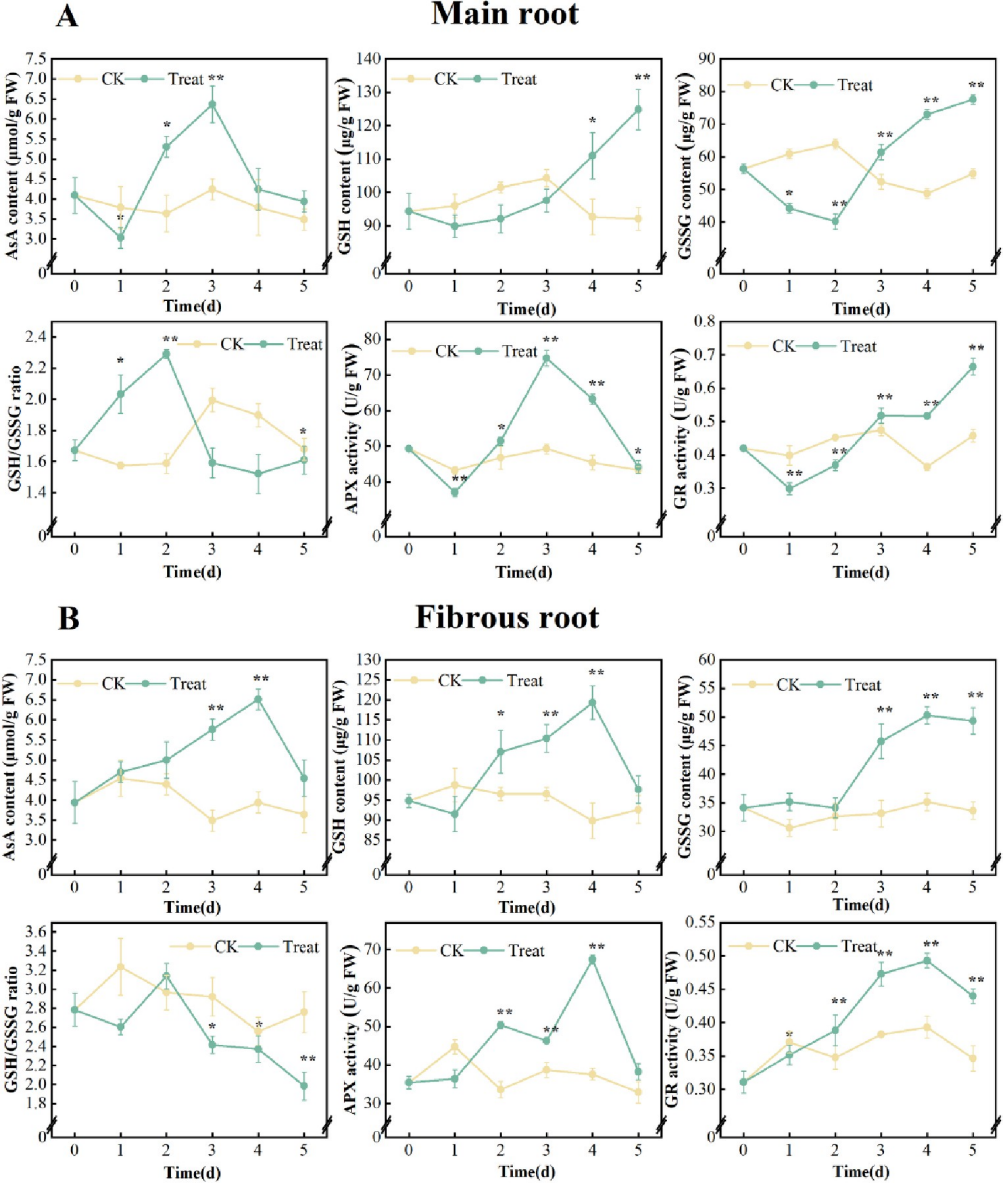
AsA, GSH, GSSH contents, ratio of GSH/GSSH and APX, GR activities in *P*. *ginseng*. (A) main root, (B) fibrous root.

The contents of AsA, GSH, and the activities of GR in ginseng fibrous root increased at first and then decreased, and all peaked on the 4^th^ day. Compared to the initial day, these levels were elevated by 65.5%, 27.3% and 58.1%, respectively. The contents of GSSG increased gradually and peaked on the 4^th^ day, with a significant increase of 47.3% compared to the 0 days. The ratio of GSH/GSSG in fibrous roots decreased. Overall, the activities of APX were increased, peaking on the 4^th^ day, with a 90.4% higher than that on the 0 days (Fig 5).

#### Effects on the activity of key enzymes of secondary metabolites

Compared with the 0 days, the activities of key enzymes relating to the ginsenoside synthesis pathway had no significant change in the ginseng main root and fibrous root in the control group, but water stress enhanced their activities during the later stage of stress.

The activities of HMGR, FPS, SS, SE and DS decreased at first and then increased under water stress, increased significantly after the 2^nd^ day (*p* < 0.01) and reached their highest levels on the 5^th^ day, with increases of 35.7%, 33.2%, 24.6%, 30.5% and 35.1%, compared with the 0 days, respectively.

**Fig 6:**
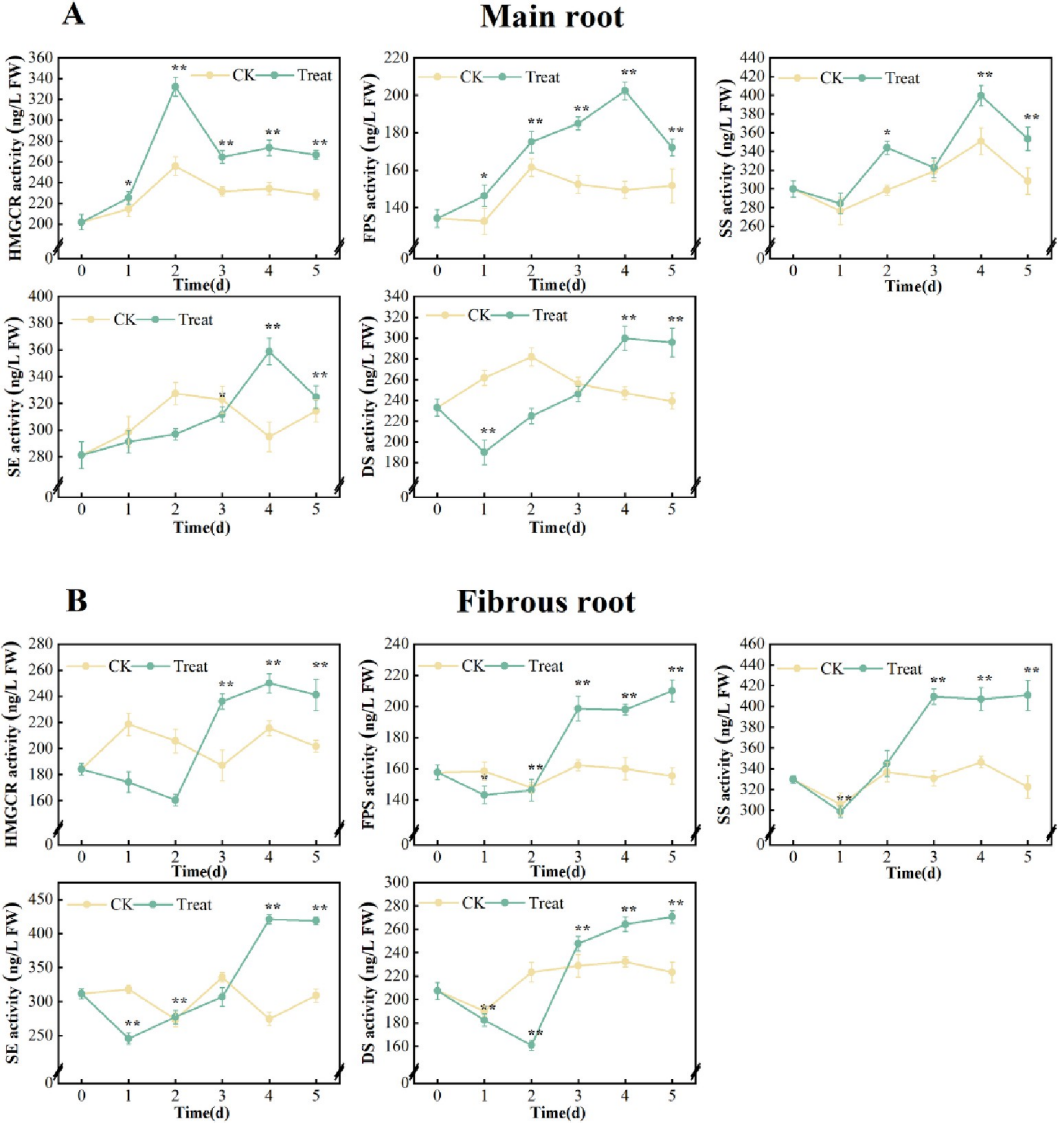
Activities of HMGCR, FPS, SS, SE and DS in *P*. *ginseng*. (A) main root, (B) fibrous root.

The activities of HMGCR, FPS, SS and SE in fibrous root exhibited similar trends. However, the activities of HMGR peaked on the 2^nd^ day, while FPS, SS and SE peaked on the 4^th^ day, with increases of 64.6%, 50.8%, 33.3% and 27.7%, respectively, compared with the 0 days. On the other hand, the activities of DS decreased at first and then increased, reaching its peak on the 4^th^ day, which was 28.6% higher than that on the 0 days (Fig 6).

The above results showed that the changes in the contents or activities of the indicators in the fibrous root occurred before the alterations in the main root.

#### Effect on the content of ginsenosides

*Monomer ginsenosides*. The levels of individual ginsenoside monomers in fresh ginseng roots varied dynamically in response to extended stress, however, the pattern of each monomer ginsenoside was not consistent.

**Fig 7:**
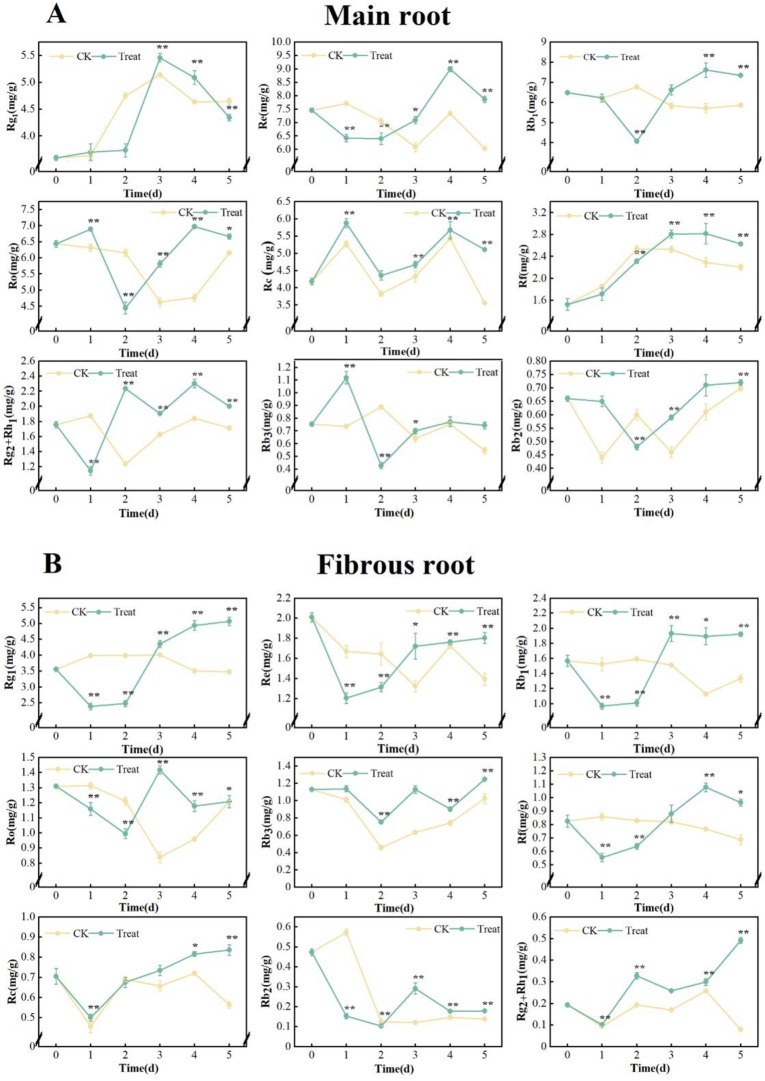
Content of monomer ginsenosides in *P*. *ginseng*. (A) main root, (B) fibrous root.

In the main root, except ginsenoside Re and Rb_2_, the contents of Rg_1_, Rf, Rg_2_+Rh_1_, Rb_1_, Rc, Ro and Rb_3_ were significantly higher than those in the control group after water stress (*p* < 0.01) at the later stages of stress. The contents of Rb_1_ and Ro peaked on the 3^rd^ day, measuring 1.93 mg/g and 1.42 mg/g, respectively. This represents an increase of 22.9% and 8.4% compared with the 0 days. The contents of Rf peaked at 1.08 mg/g on the 4^th^ day, which was 30.1% higher than that on the 0 days. The contents of Rg_1_, Rg_2_+Rh_1_, Rc and Rb_3_ reached their highest level on the 5^th^ day, measuring 5.07 mg/g, 0.49 mg/g, 0.84 mg/g and 1.25 mg/g, respectively, increased by 42.4%, 157.9%, 18.3% and 10.6% compared with the 0 days (Fig 7A).

In the fibrous root, the contents of Rc and Rb_3_ peaked on the first day, were 5.87 mg/g and 1.12 mg/g, respectively. This represents a rise of 40.4% and 45.5% compared with the 0 days. The contents of Rg_1_ peaked at 5.46 mg/g on the 3^rd^ day, which was 52.1% higher than that on the 0 days. The contents of Re, Rf, Rg_2_+Rh_1_, Rb_1_ and Ro peaked on the 4^th^ day, were 8.99 mg/g, 2.82 mg/g, 2.30 mg/g, 7.61 mg/g and 6.98 mg/g, respectively. These concentrations increased by 20.5%, 84.3%, 30.7%, 17.3% and 8.6% compared with the 0 days. The contents of Rb_2_ peaked at 0.72 mg/g on the 5^th^ day, which was 9.1% higher than that on the 0 days (Fig 7B).

Rg_1_, Re and Rb_1_ are the main indicators to evaluate the quality of ginseng. The treated main root showed the highest improvement on the 5^th^ day. Rg_1_+Re and Rb_1_ increased by 42.4% and 21.0%, respectively. Additionally, Rb_3_, Rf, Rc, and Rg_2_+Rh_1_ also surged by 10.6%, 15.7%, 18.3%. and 157.9%, respectively. The treated ginseng fibrous root showed the highest improvement on the 4^th^ day. Rg_1_, Re and Rb_1_ increased by 41.8%, 20.5% and 17.3%, respectively. Additionally, Ro, Rc, Rf, Rg_2_+Rh_1_, and Rb_2_ also increased by 8.6%, 35.6%, 84.3%, 30.7%, and 7.6%, respectively.

**Fig 8:**
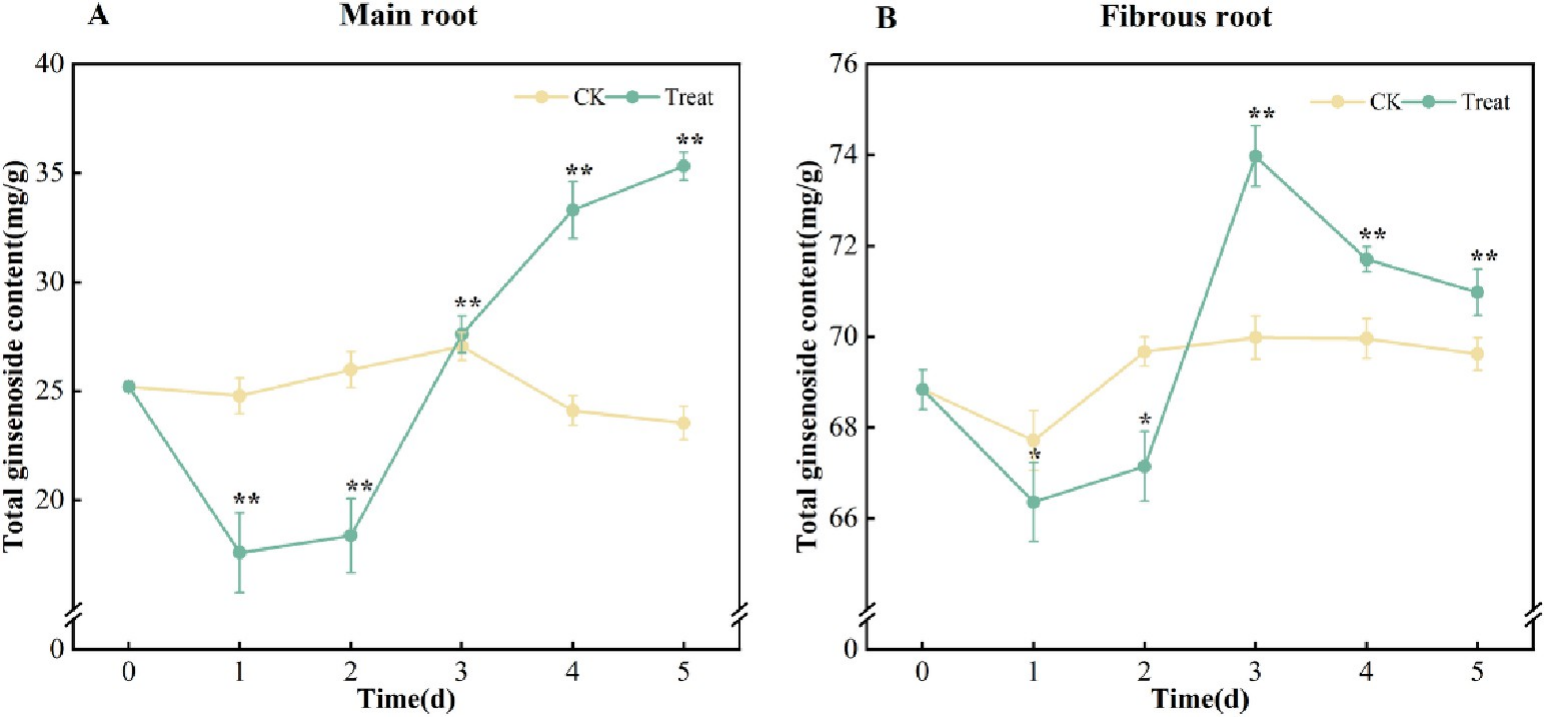
Contents of total ginsenosides in *P*. *ginseng*. (A) main root, (B) fibrous root.

*Total ginsenosides*. The total ginsenosides of the ginseng fresh roots were calculated by the regression equation, Y = 0.0043X+0.0247 (R^2^ = 0.999), where Y stood for absorbance and X for the Re concentration (mg/ml). Compared with the 0 days, the control group showed minimal variation in the total ginsenosides, while the stressed roots exhibited varying degrees of growth. In the main root, the total ginsenosides decreased first and then increased, with the highest content of 35.1 mg/g on the 5^th^ day, which was 40.1% higher than that on the 0 days. The fibrous root exhibited a decreasing-increase-decrease course, and reached its highest point at 73.97 mg/g on the 3^rd^ day, which was 7.5% higher than that on the 0 days (Fig 8).

#### Effects of Water stress on 1,3-DPG content and PEPC activity of *P*. *ginseng*

When comparing the 0 days, there was no significant change in the levels of 1,3-DPG contents and PEPC activities in the main root and fibrous root of the control group. However, these levels rose under water stress.

**Fig 9:**
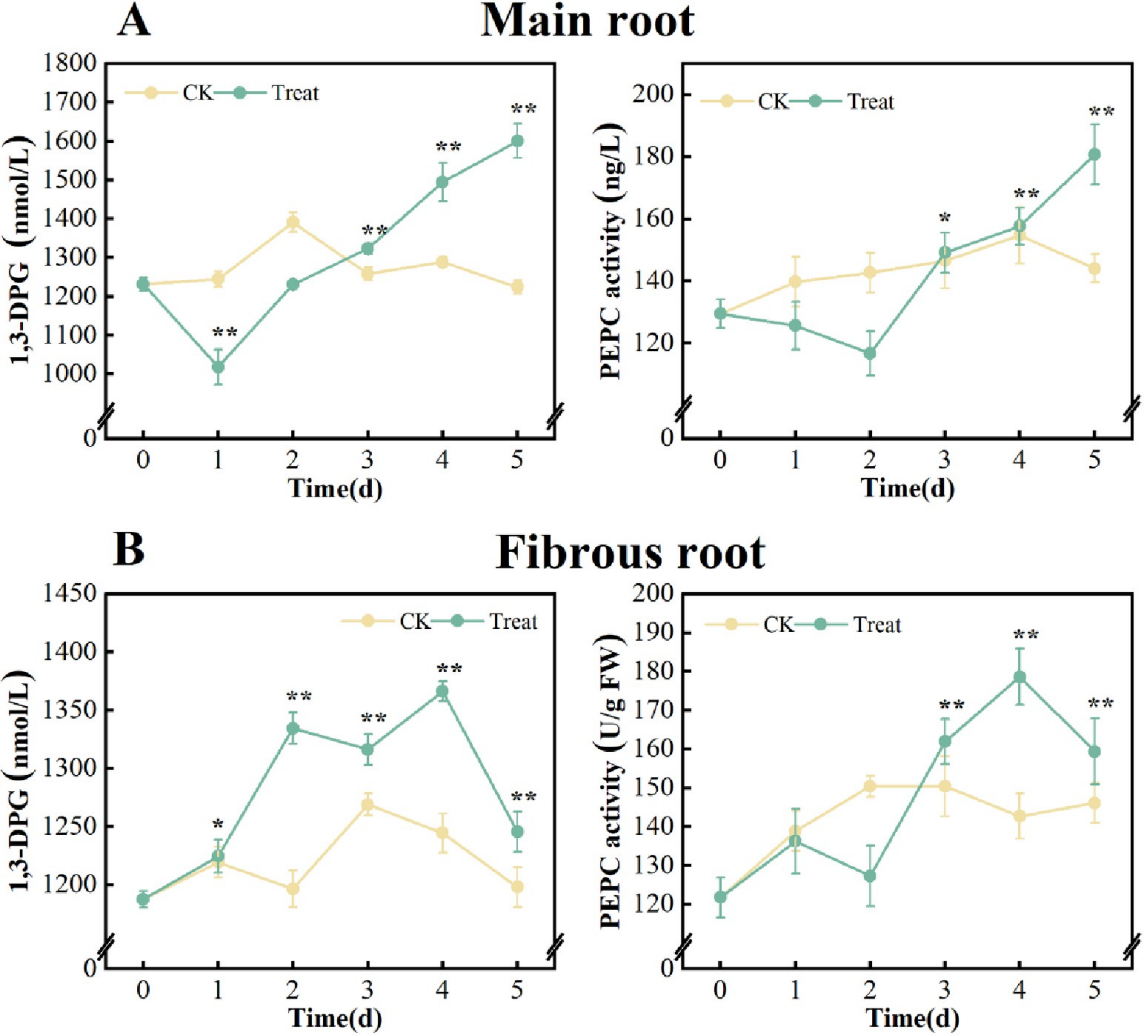
1,3-DPG contents and PEPC activities in *P*. *ginseng*. (A) main root, (B) fibrous root.

The contents of 1,3-DPG in the main root decreased first and then increased during the stress, peaking on the 5^th^ day, with a 29.9% increase compared to that on the 0 days. PEPC activities increased gradually and also peaked on the 5^th^ day, with a 39.6% increase compared to the 0 days. However, the fibrous root observed an initial increase followed by a subsequent drop, reaching its highest point on the 4th day. The increase was measured at 15.0% and 46.7% compared to the first measurement on day 0 (Fig 9).

#### Principal componnent analysis

**Fig 10:**
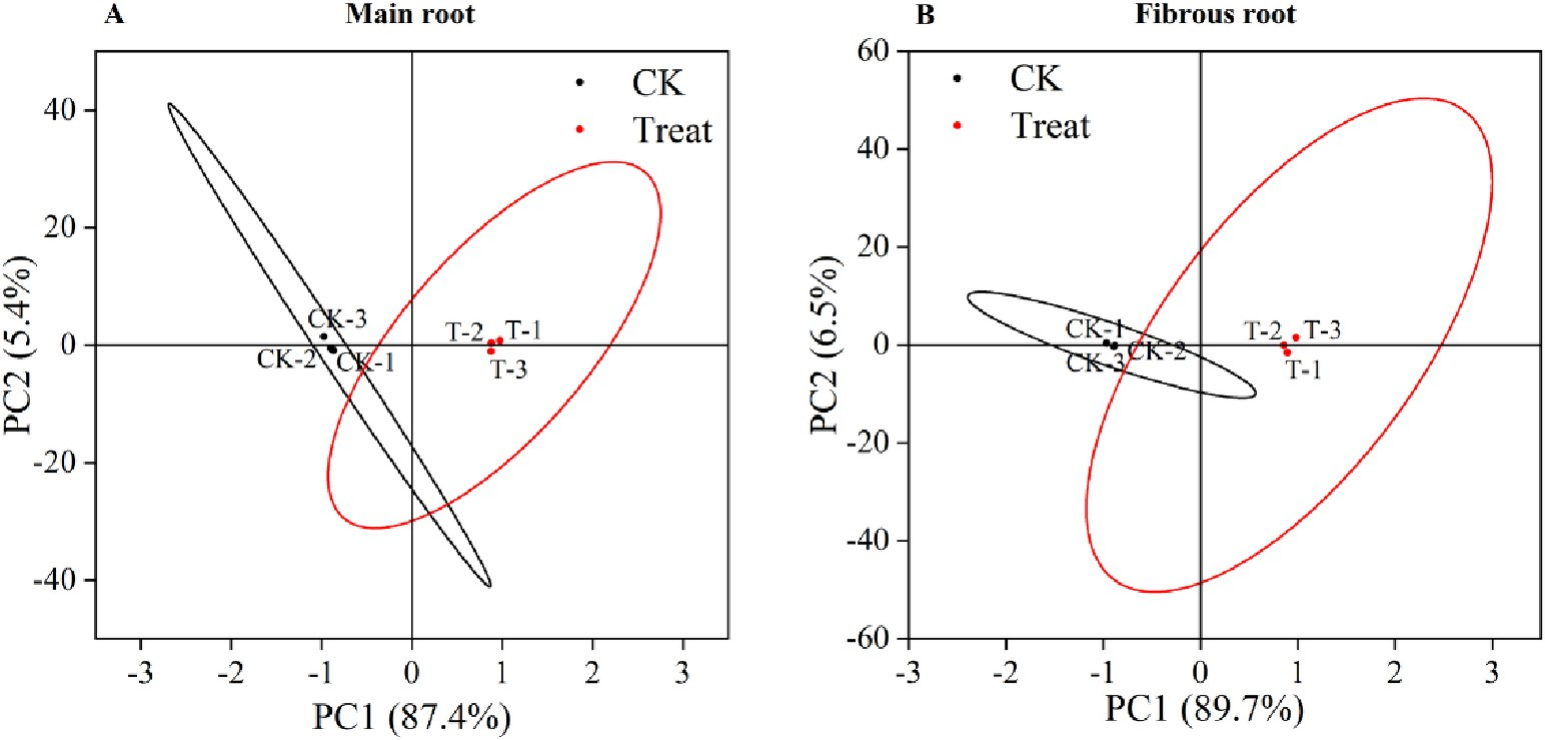
PCA of *P*. *ginseng*. (A) main root, (B) fibrous root.

**Fig 11:**
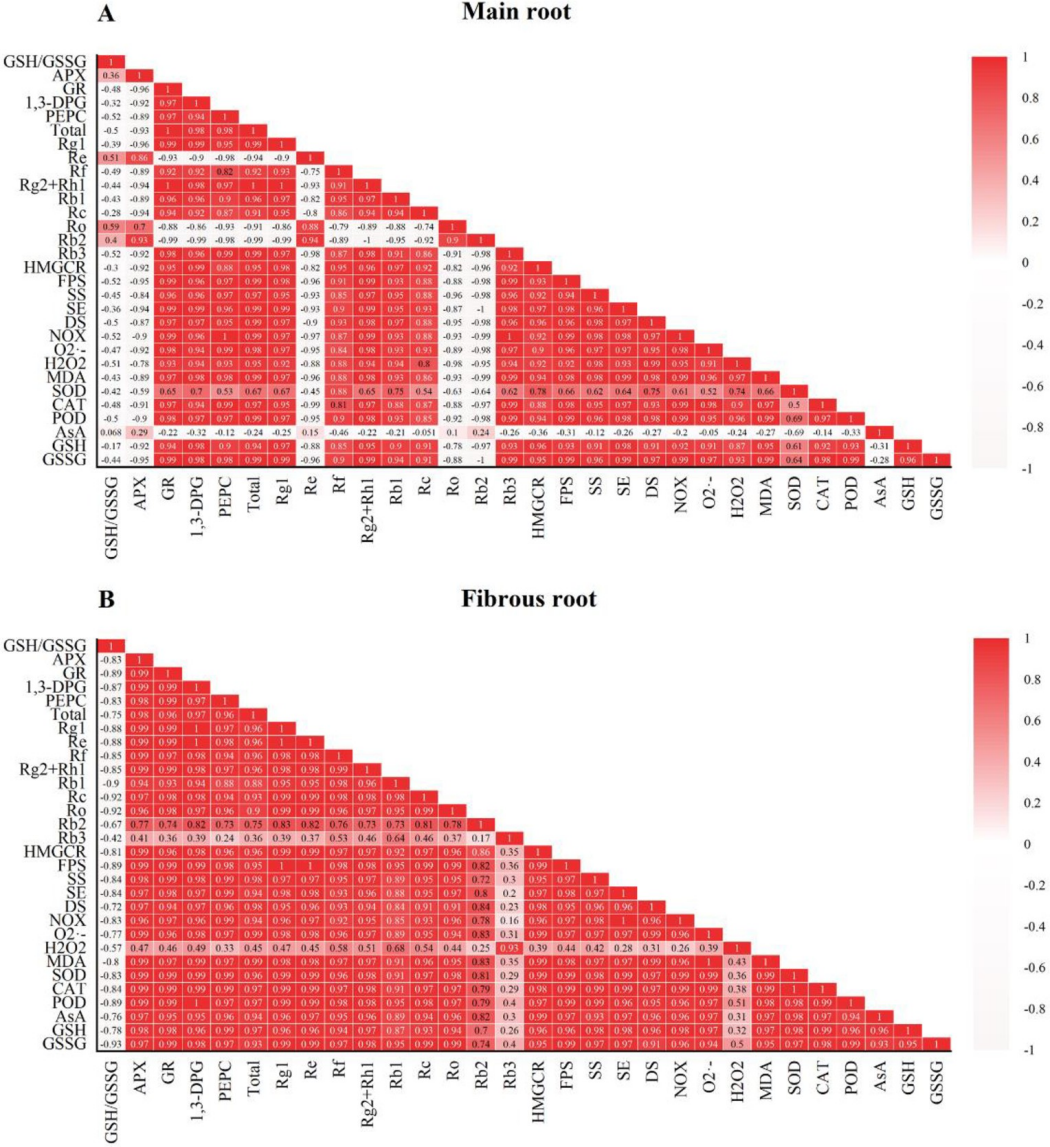
Pearson correlation chart. (A) main root, (B) fibrous root.

The results of Principal Component Analysis (PCA) showed that the ginseng control and stress groups were separated, indicating that there were significant differences between the control and stress groups and a high level of reproducibility. The cumulative contribution of the two principal components (PC1 and PC2) in the main root was 92.8%, with PC1 accounting for 87.4% and PC2 accounting for 5.4%. The percentage of fibrous roots was 96.2%, with PC1 accounting for 89.7% and PC2 accounting for 6.5% (Fig 10). The correlation chart displayed the positive or negative correlations among the analyzed parameters (Fig 11). The degree of correlation between analyzed parameters is differentiated based on Pearson’s value. Correlation coefficients with absolute values greater than 0.8 are classified as strong correlations, those less than 0.3 are classified as weak correlations, and others are moderate correlations.

#### Pharmacological effects verification

**Fig 12:**
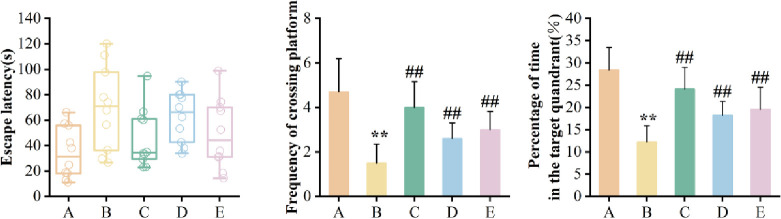
Results of Morris water maze experiment in mice. (A) blank control group. (B) model group. (C) positive control group. (D) ginseng main root group on the 0^th^ day. (E) stressed ginseng main root group on the 5^th^ day. All values were expressed as Mean ± S.D. (n = 10). **p < 0.01 vs. blank control group (A); ##*p* < 0.01 vs. model group (B).

*Morris water maze experiment*. Compared with the ginseng main root group on the 0^th^ day, the escape latency of the stressed ginseng main root on the 5^th^ day was shortened by 21.4% (*p* = 0.228), the number of times crossing the original platform and the residence time of the target area was increased by 0.4 (*p* = 0.255) and 7.3% (*p* = 0.486), respectively (Fig 12), indicating that water-treated ginseng has the potential to partially cure memory impairment in rats.

**Fig 13:**
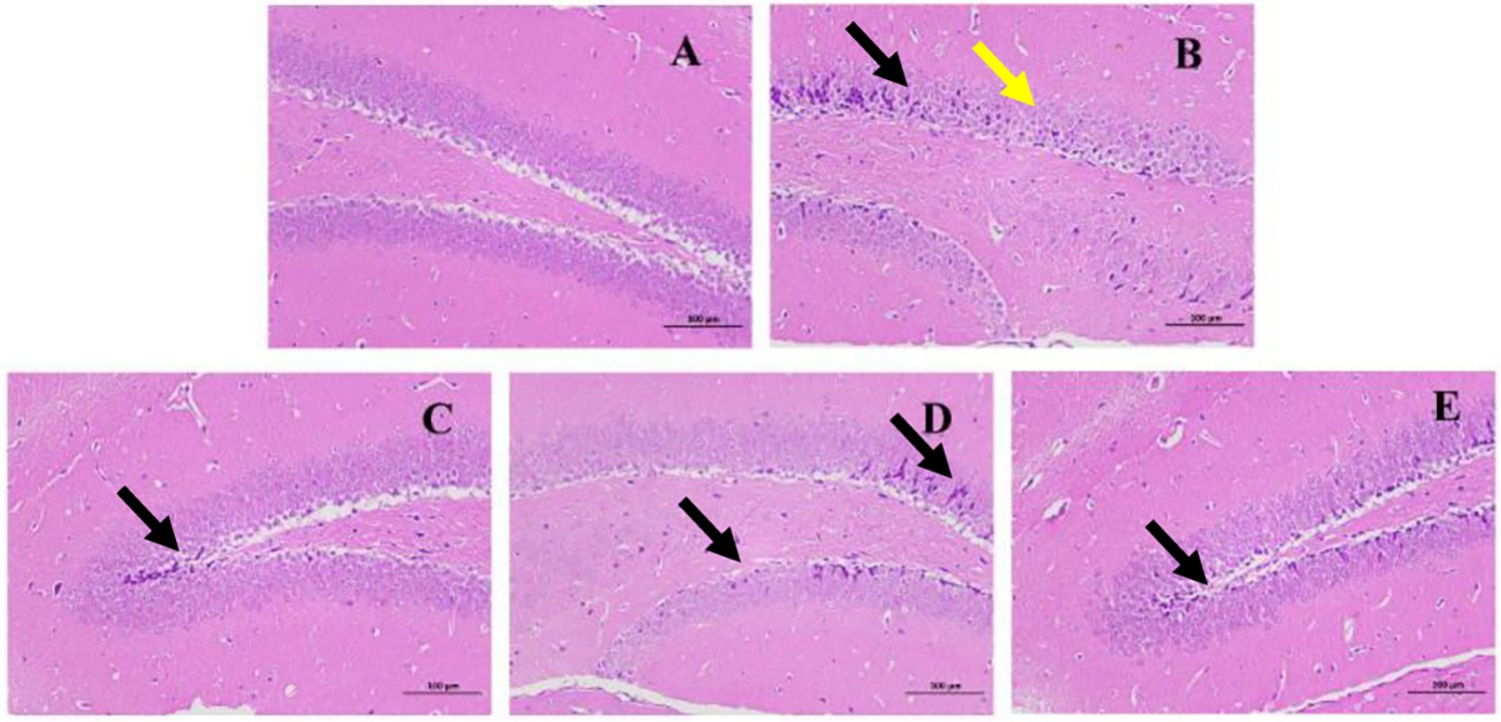
Mice brain tissues stained with H&E dye kits (200×). (A) blank control group. (B) model group. (C) positive control group. (D) main root group on the 0^th^ day. (E) stressed the main root group on the 5^th^ day.

*Histopathological staining*. Compared with the model group, the degree of lesions in the hippocampus of mice in the positive control group, main root group on the 0^th^ day and stressed main root group on the 5^th^ day were significantly reduced. The hippocampus lesion degree of the stressed group on the 5^th^ day was less severe than that on the 0^th^ day (Fig 13).

**Fig 14:**
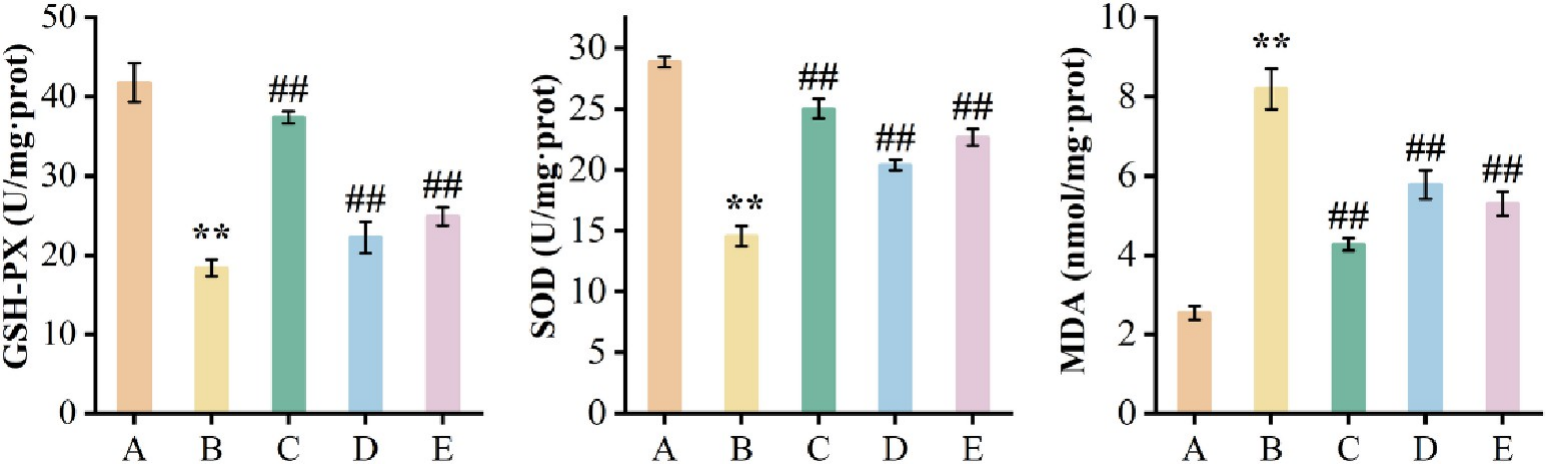
GSH-PX, SOD activities and MDA contents in mice brain tissues. (A) blank control group. (B) model group. (C) positive control group. (D) main root group on the 0^th^ day. (E) stressed the main root group on the 5^th^ day. All values were expressed as Mean ± S.D. (n = 10). **p < 0.01 vs. blank control group (A); ##*p* < 0.01 vs. model group (B).

*Oxidation-associated indexes in mice*. Compared with the model group, the GSH-PX activity in the positive control group, main root group on the 0^th^ day, and stressed main root group on the 5^th^ day were increased by 103.4%, 21.0% and 35.5% (*p* < 0.01). Similarly, SOD activities increased by 71.8%, 40.0% and 55.7% (*p* < 0.01), respectively. On the other hand, MDA contents decreased by 47.9%, 29.5% and 35.4%, respectively (*p* < 0.01). Compared with the main root group on the 0^th^ day, the activities of GSH-PX and SOD in the stressed main root group on the 5^th^ day were increased by 12.0% (*p* = 0.119) and 11.2% (*p* = 0.009), respectively, while the content of MDA was decreased by 8.3% (*p* = 0.155) (Fig 14). Although P > 0.05, it also showed that the quality of the medicine was significantly improved.

## Discussion

Plant stress often leads to a burst of ROS, which in turn triggers an increase in antioxidant substances such as antioxidant enzymes and secondary metabolites [29]. The active components of medicinal plants are usually secondary metabolites, which play a crucial role in the plant’s ability to adapt to its environment. Therefore, the process by which plants adapt to their environment is closely linked to the development of high-quality therapeutic ingredients.

### Effects of water stress on the contents of ROS and MDA in ginseng

Water stress leads to a gradual decrease in oxygen concentration and an imbalance in redox potential in *P*. *ginseng*. This, in turn, leads to a decrease in photosynthetic electron transport and PSⅡefficiency, induces the surge of H_2_O_2_-centered ROS in plants, causes membrane peroxidation, damage the cell membrane and increases membrane permeability [30–32]. Fig 1 shows that O_2_^·-^and H_2_O_2_ in the main root of *P*. *ginseng* initially decreased in the early stage of water stress. The hypoxia caused by water stress can be attributed to the destruction of the mitochondrial site of ROS generation, as the plant undergoes both aerobic and anaerobic respiration. O_2_^·-^ contents peaked on the 3^rd^ day and then decreased, while H_2_O_2_ increased gradually after the 2^nd^ day and peaked on the 5^th^ day. This pattern suggests that to maintain a balance between oxidation and antioxidants, plants initiate their own antioxidant system to eliminate excessive ROS, albeit with a certain delay in activation. In general, O_2_^·-^ and H_2_O_2_ in fibrous roots demonstrate a higher sensitivity to water stress, possibly because the fibrous roots are more susceptible to water uptake.

NOX located in the plasma membrane, is the main source of ROS [33], which catalyzes the transfer of electrons from NADPH to O_2_, generating O_2_^·-^ [34]. Previous studies have found that stress can enhance NOX activity in ginseng cell membranes and promote H_2_O_2_ production in ginseng suspension cells [35,36]. The identical behavior was observed in fresh ginseng root in this study. In the main root, the activity of NOX increased linearly on the 2^nd^ day of stress and peaked on the 5^th^ day. NOX activity in fibrous roots increased dramatically right after stress, peaked on day 4, and gradually decreased, although still staying considerably elevated compared to day 0 (Fig 2). It could be attributed to the relatively large surface area of fibrous roots, which makes them more vulnerable to water stress. This indicated that excessive water could stress the fresh roots of ginseng. The MDA in ginseng gradually increased, which correlated with the change in ROS content. This observation further confirmed that ROS is responsible for the damage inflicted on fresh ginseng roots (Fig 3).

### Effects of water stress on main antioxidant enzymes

The ROS produced in plants interact with other signal molecules to activate antioxidant mechanisms for regulation [37]. SOD, CAT, POD and APX are inductive enzymes. Their activities are enhanced when ROS levels increase within a certain amount. Conversely, their activity decreases when ROS levels fall. This mechanism plays a crucial role in defending plant cells against ROS injury. SOD directly converts O_2_^·-^ into H_2_O_2_ and H_2_O, whereas POD and CAT reduce ROS damage by generating H_2_O from H_2_O_2_ through catalytic catabolism [38]. In this study, SOD in the ginseng main root showed a decreasing trend at the beginning of stress. A large number of studies have shown that SOD activity decreases in plants at the initial stage of stress [39–41]. Although the mechanism is unclear, the decrease in SOD activity in the early stage can promote the accumulation of ROS and lead to an increase in antioxidants, ultimately improving adaptability. In the middle and late stages of stress, SOD, CAT and O_2_^·-^ initially increased and then decreased, while POD and H_2_O_2_ continued to rise (Figs 1 and 4). This suggests that ginseng exhibits a protective effect against water stress in plants. Nevertheless, as the duration of stress increased, there was a rapid accumulation of H_2_O_2_, accompanied by a general drop in the activity of antioxidant enzymes. This led to an increase in membrane lipid peroxidation and increased MDA content (Fig 3). Thus, the enhancement of antioxidant enzyme activity in response to water stress was not sufficient to scavenge excessive ROS and repair oxidative damage.

### Effect of water stress on AsA-GSH cycle

The AsA-GSH cycle clears H_2_O_2_ driven by NADPH and exists in many plant subcellular compartments. Its main function is to convert AsA and GSH into their oxidative form in order to eliminate ROS and maintain intracellular redox balance. GSH content and GSH/GSSG ratio are the key factors to determine the operation efficiency of this cycle [42]. High GSH/GSSG ratio can inhibit the formation of intermolecular disulfide bonds, form a reduction environment in cells, and maintain the proper structure and activity of protein molecules [43]; conversely, low levels of GSH/GSSG ratio trigger the activation of resistance gene signals in response to adverse environmental conditions [42]. APX utilizes AsA as an electron donor to catalyze the decomposition of H_2_O_2_ into H_2_O and O_2_ [44], whereas GR utilizes NADPH as an electron donor to reduce GSSG to GSH [45]. The activity of both enzymes serves as a key indicator of plant stress tolerance [46].

In this study, a similar trend of AsA content and the APX activity in the main root and fibrous root of ginseng was observed. These values were significantly higher than those of the control in the early and middle stages of stress (Fig 5). AsA is a crucial low-molecular-weight, water-soluble, non-enzymatic antioxidant. Under water stress conditions, AsA is the primary molecule utilized to supply electrons to APX, facilitating the detoxification of excess H₂O₂ in plants. GSH is a low-molecular water-soluble mercaptan compound that exerts its antioxidant effect by directly scavenging ROS or indirectly scavenging H_2_O_2_ through the AsA-GSH cycle. In this study, GSH, GSSG and GR all showed a steady rising trend in the later stage of stress, indicating a regular change in response to water stress (Fig 5). However, the effect was relatively slight in the early stage of stress. These findings suggest that GR did not participate in ROS clearance initially, but its function was replaced by other antioxidant enzymes such as APX over this period. However, the continuous accumulation of H_2_O_2_ induced the high activity of GR. Still, it did not impede the regeneration of GSH. As a result, the cycle of GR-GSH remained reduced, effectively eliminating the excessive ROS generated in the cells. The decrease of GSH/GSSG ratio may be attributed to the reduced rate of GSH regeneration relative to GSSH or lack of NADPH in cells. At this point, the balance of ROS in the cell was disrupted, which further led to the blockage of the AsA cycle, ultimately leading to cell damage.

Previous investigations on *Vigna radiata* L roots have revealed a similar outcome in terms of APX activity, as well as a further reduction in the efficiency of the ASA-GSH cycle [47].

### Effects of water stress on the synthesis of metabolites

Ginsenosides not only directly scavenge ROS, but also indirectly increase the activities of antioxidant enzymes such as SOD and CAT, acting as membrane stabilizers and ROS scavengers [48]. The biosynthesis of ginsenosides consists of three stages, including more than 20 consecutive enzymatic reactions: (1) In the stage of terpene precursor biosynthesis, HMGCR is the key rate-limiting enzyme. When the HMGCR level increases, it promotes the MVA pathway, which in turn promotes ginsenoside synthesis [49]. (2) During the prolonged stage of parent nucleotide C skeleton, FPS, SS and SE are the key enzymes. (3) In the skeleton structuring of parent nucleotide and its post-modification stage, DS, β-AS, CYP450 and GT are key enzymes [50]. The activities of DS and GT were positively correlated with ginsenoside content [51]. The results showed a significant correlation (*p* < 0.05) between soil water potential and the expressions of key ginsenoside synthesis enzymes, namely HMGCR, SS, β-AS, DS and CYP716A47 genes. These results indicate that increasing soil water potential can significantly promote the expression of key enzyme genes in ginseng roots [52].

The accumulation of monomer ginsenosides and total ginsenosides in the main and fibrous root of *P*. *ginseng* showed a time-dependent effect on water stress in this study. The activities of HMGCR and SE in the main root of *P*. *ginseng* increased significantly on the 3^rd^ and 4^th^ day after stress (*p* < 0.01), respectively, and peaked on the 4^th^ day. On the other hand, the activities of FPS, SS and DS all markedly increased on the 3^rd^ day (*p* < 0.01) and peaked on the 5^th^ day. The activity of HMGR in fibrous root significantly climbed and peaked on the 2^nd^ day, and the activity of FPS, SE, SS and DS all reached a peak on the 4^th^ day (Fig 6). The results showed that the activities of five key enzymes in the ginsenoside biosynthesis pathway exhibited a consistent increase during the whole stress period. This increase in enzyme activity is likely to promote the biosynthesis of ginsenosides.

As a result of the changes in the activity of the five ginsenosides synthase mentioned above, the content of monomer ginsenosides in ginseng also changed dramatically. These changes were generally in line with the activity of DS, which is downstream of ginsenosides synthesis. Among the ten monomers, tetracyclic triterpenoid ginsenosides Rg_1_, Re, Rf, Rg_2_ and Rh_1_ belong to protopanaxatriol type (PPT), Rb_1_, Rc, Rb_2_ and Rb_3_ belong to protopanaxadiol type (PPD), and Ro belongs to oleanane type of pentacyclic triterpenoid ginsenosides. In the main root, the contents of all monomer ginsenosides except for ginsenosides Re and Rb_2_ increased to different degrees. The highest increase was observed in the levels of ginsenoside Rg_2_+Rh_1_ in the main root, which reached a maximum of 157.9% on the 5^th^ day. This is followed by a 42.4% increase in the levels of Rg_1_. All 10 monomer ginsenosides in the fibrous roots were increased and all showed highly significant changes from day 0 (*p* < 0.01). Among the fibrous roots on day 4, Rf was the most elevated, at 84.3%, followed by Rg_2_+Rh_1_ at 30.7% (Fig 7). PPT ginsenosides Rg_1_, Rf and Rg_2_+Rh_1_ are more sensitive to water stress than PPD ginsenosides Rb_1_, Rc and Rb_3_, which may be attributed to the extra c-3 hydroxyl group. There was no obvious change in Re in the ginseng main root, possibly because the main root experienced mild stress that was insufficient for Rg_1_ to combine with rhamnose to form Re. The hemicellulose and pectin of plant cell walls contain a large amount of L-arabinose [53], and the 20th position of ginsenoside Rb_2_ is connected to arabinopyranose. Therefore, it is speculated that Rb_2_ in ginseng main root is consumed as a supplement to the cell wall during stress. It was recently reported that UDP-arabinose mutase often catalyzes the formation of UDP-arabinosylfuranose from UDP-arabinosylpyranose in plants [54]. It appears to clarify the notable rise in ginsenoside Rc, which is linked to arabofuranose at the C-20 position, although additional experimental confirmation is required. The content of total saponins in the main root of *P*. *ginseng* increased continuously and peaked on the 5^th^ day. In contrast, the content of total saponins in fibrous root increased initially, peaked on the 3^rd^ day, and then decreased. The rate of increase in fibrous root was significantly lower than in the main root (Fig 8). The fibrous root is more delicate than the main root, and it responds quickly to stress, which hinders the production of total ginsenosides. The significant decrease in its content may be attributed to the ingestion of ginsenosides as defensive compounds [55].

Glycolysis and tricarboxylic acid cycle are essential energy and raw material pathways for plants to synthesize most secondary metabolites. 1,3-DPG is an important intermediate product of glycolysis and the Calvin cycle, serving as an indicator of glucose catabolism [56]. PEPC plays an important role in regulating the operation of the tricarboxylic acid cycle. It can catalyze the reaction of phosphoenolpyruvate with CO_2_ to form oxaloacetic acid (the starting material of the tricarboxylic acid cycle), and the subsequent synthesis of malic acid and its derivatives [57]. In this study, the 1,3-DPG content and PEPC activity in fresh roots of *P*. *ginseng* rose to different extents under stress (Fig 9). It shows that water stress can promote the conversion of sugar in fresh ginseng root into subsequent material, energy metabolism and ginsenosides., This indicates that ginsenosides are derived from sugars rather than other active saponins, resulting in a significant increase in the content of various ginsenosides.

**Fig 15:**
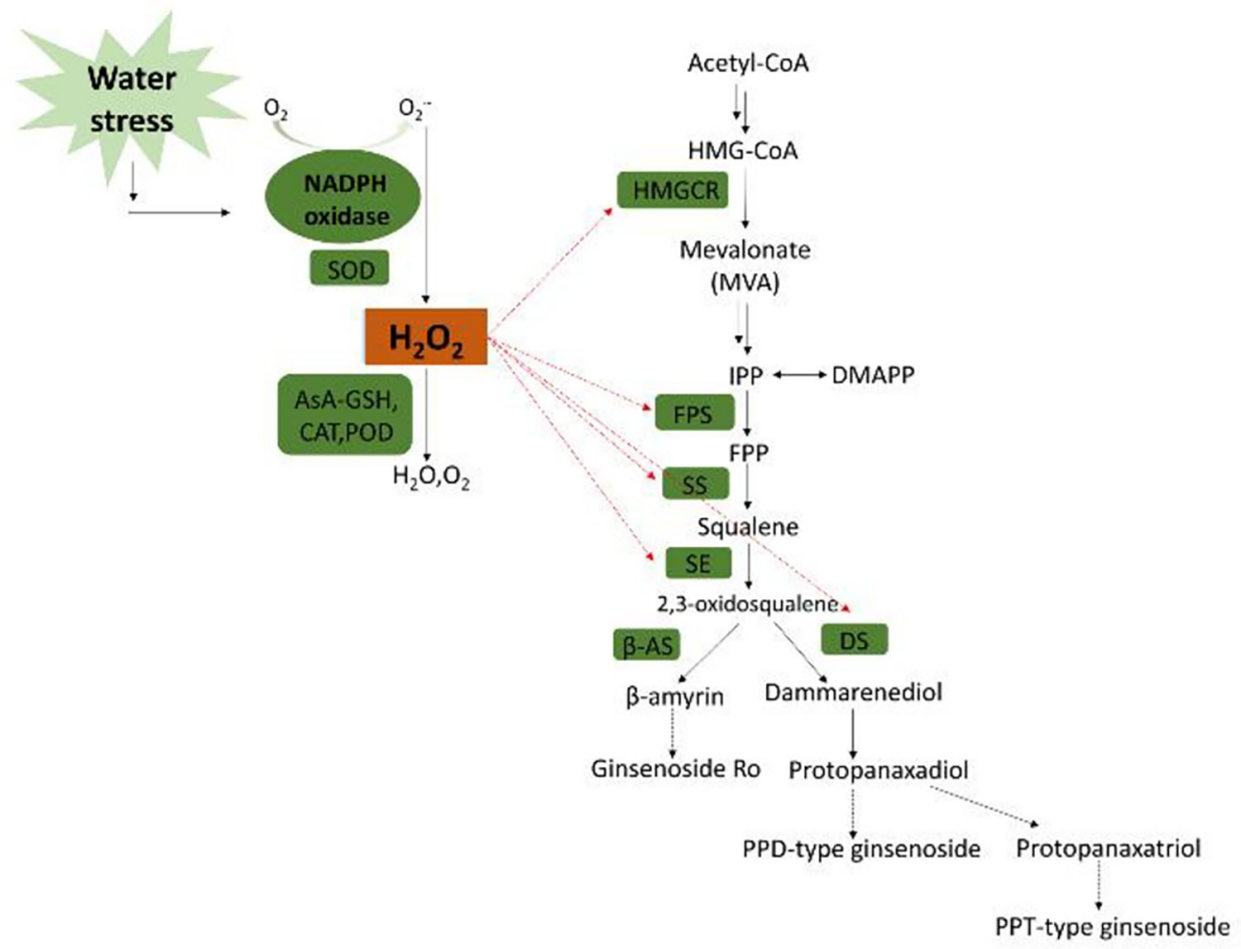
ROS promote ginsenoside biosynthesis under water stress. Green boxes represent enzymes and red dashed arrows represent the relationship between ROS and the activities of key enzymes of the ginsenoside synthesis pathway.

Taken together, water stress can induce fresh ginseng roots to up-regulate the activities of antioxidant enzymes, enhance AsA-GSH cycle metabolism and trigger the accumulation of secondary metabolites. This helps reduce the negative effects of water stress on themselves. Fig 15 illustrates the mechanism via which ROS enhances the production of ginsenoside under conditions of water stress.

### Validation of pharmacodynamics

The content of active ingredients is an important indicator for evaluating the quality of herbal medicine. However, herbal medicines contain a wide range of active components with varying levels and activities, making it difficult to evaluate the quality based on chemical component content [25]. Ginsenosides, especially Rg_1_ and Rg_2_ have anti-aging effect [28,58,59]. The D-galactose-induced subacute aging model has been widely used in experimental anti-aging studies because of its similarity to natural aging [60]. In this study, the ginseng main root on the 5^th^ day showed better efficacy (Fig 11). The escape latency was reduced by 21.4% (*p* = 0.228), the number of times crossing the original platform and the residence time in the target area increased by 0.4 times (*p* = 0.255) and 7.3% (*p* = 0.486), respectively, compared with the 0 days. It suggests that ginseng can intervene in the aging process and significantly antagonized the impairment of learning memory function in mice by D-galactose. SOD and GSH-PX have significant functions in antioxidant, anti-aging and anti-injury. The MDA content is positively correlated with the formation of free radicals. In this study, the activities of GSH-PX and SOD on the 5^th^ day were higher than those on the 0 days. Specifically, SOD increased by 11.2% (*p* = 0.009). The MDA showed a decreasing trend, although there was no significant difference between them (*p* = 0.155) (Fig 14). It suggests that ginseng can effectively attenuate D-galactose-induced oxidative damage in mice brain tissues by activating antioxidant enzyme activities, it is consistent with the study of Hao *et al* [61]. Combined with the results of morphological staining (Fig 13), the present study showed that water stress significantly improved the quality of ginseng.

## Conclusions

Short-term water stress can trigger a physiological state of oxidative stress in ginseng fresh roots, stimulate the antioxidant defense system in ginseng, and lead to increased synthesis of secondary metabolites. In the main root, the ginsenosides Rg_1_+Re, Rb_1_, Rf, Rg_2_+Rh_1_, Rc and Rb_3_ increased by 42.4%, 21.0%, 15.7%, 157.9%, 18.3% and 10.6%, respectively, and the total ginsenosides by 40.1%. In fibrous roots, Rg_1_, Re, Rb_1_, Rf, Rg_2_+Rh_1_, Rc, Ro and Rb_2_ increased by 41.8%, 20.5%, 17.3%, 84.3%, 30.7%, 35.6%, 8.6% and 7.6%, respectively, the total ginsenosides increased by 4.2%. The activities of antioxidant enzymes were also enhanced, and they acted synergistically with secondary metabolites to minimize the damage caused by ROS. The effectiveness of the main root of ginseng was somewhat increased on the 5th day due to improved secondary metabolites. In summary, the quality of ginseng could be improved by water stress. Ginseng has a narrow ecological amplitude due to the lack of flavonoids and prefers humidity to waterlogging. Therefore, this study offers novel insights into ginseng’s ability to withstand water stress and its impact on quality formation.

## Supporting information

S1 Data (XLSX)

## References

[1] Mittler R, Blumwald E. The roles of ROS and ABA in systemic acquired acclimation. Plant Cell. 2015; 27(1): 64–70. doi: 10.1105/tpc.114.133090 25604442 PMC4330577

[2] Noctor G, Mhamdi A, Foyer C H. The roles of reactive oxygen metabolism in drought: not so cut and dried. Plant Physiol. 2014; 164(4): 1636–1648. doi: 10.1104/pp.113.233478 24715539 PMC3982730

[3] Czarnockaa W, Stanisław K. Friend or foe? Reactive oxygen species production, scavenging and signaling in plant response to environmental stresses. Free Radical Bio Med. 2018; 122: 4–20. doi: 10.1016/j.freeradbiomed.2018.01.011 29331649

[4] You J, Chan ZL. ROS regulation during abiotic stress responses in crop plants. Front Plant Sci. 2015; 6: 1092. doi: 10.3389/fpls.2015.01092 26697045 PMC4672674

[5] Meng XC, Deng DQ, Du HW, Guan Y. Scientific connotation of high-quality genuine medicinal materials. Chinese Traditional and Herbal Drugs. 2023; 54(3): 939–947.

[6] Wink M. Introduction: biochemistry, physiology and ecological functions of secondary metabolites. Annual plant reviews volume 40: Biochemistry of plant secondary metabolism. 2010; 1–19. doi: 10.1002/9781444320503.ch1

[7] Edreva A, Velikova V, Tsonev T, Dagnon S, Gürel A, Aktaş L, et al. Stress-protective role of secondary metabolites: diversity of functions and mechanisms. Gen. Appl. Plant Physiol. 2008; 34(1–2): 67–78.

[8] Khokon MAR, Jahan MS, Rahman T, Hossain MA, Muroyama D, Minami I, et al. Allyl isothiocyanate (AITC) induces stomatal closure in Arabidopsis. Plant Cell Environ. 2011; 34(11): 1900–1906. doi: 10.1111/j.1365-3040.2011.02385.x 21711355

[9] Lee BR, Li LS, Jung WJ, Jin YL, Avice JC, Ourry A, et al. Water deficit-induced oxidative stress and the activation of antioxidant enzymes in white clover leaves. BIOL PLANTARUM. 2009; 53(3): 505–510. doi: 10.1007/s10535-009-0091-2

[10] Dong L, Wang Y, Lv J, Zhang H, Jiang N, Lu C, et al. Memory enhancement of fresh ginseng on deficits induced by chronic restraint stress in mice. Nutr. Neurosci. 2019; 22(4): 235–242. doi: 10.1080/1028415X.2017.1373928 28911273

[11] Chen XC, Chen Y, Zhu YG, Fang F, Chen LM. Protective effect of ginsenoside Rg_1_ against MPTP-induced apoptosis in mouse substantia nigra neurons. Acta Pharmacol. Sin. 2002, 23(9): 829–834. 12230953

[12] Guilak F, Nims RJ, Dicks A, Wu CL, Meulenbelt I. Osteoarthritis as a disease of the cartilage pericellular matrix. Matrix Biol. 2018; 71: 40–50. doi: 10.1016/j.matbio.2018.05.008 29800616 PMC6146061

[13] Kang OJ. Antioxidant activities of various solvent extracts from Ginseng (Panax ginseng CA Meyer) leaves. Journal of Food Science and Nutrition. 2011; 16(4): 321–327. doi: 10.3746/jfn.2011.16.4.321

[14] Chung IM, Lim JJ, Ahn MS, Jeong HN, An TJ, Kim SH. Comparative phenolic compound profiles and antioxidative activity of the fruit, leaves, and roots of Korean ginseng (Panax ginseng Meyer) according to cultivation years. J. Ginseng Res. 2016; 40(1): 68–75. doi: 10.1016/j.jgr.2015.05.006 26843824 PMC4703808

[15] Kim J S. Investigation of phenolic, flavonoid, and vitamin contents in different parts of Korean ginseng (Panax ginseng C.A Meyer). Preventive nutrition and food science. 2016; 21(3): 263. doi: 10.3746/pnf.2016.21.3.263 27752503 PMC5063212

[16] Ru W, Wang D, Xu Y, He X, Sun YE, Qian L, et al. Chemical constituents and bioactivities of Panax ginseng (CA Mey.). Drug Discov Ther. 2015; 9(1): 23–32. doi: 10.5582/ddt.2015.01004 25788049

[17] Qi H, Li L, Ma H. Cellular stress response mechanisms as therapeutic targets of ginsenosides. Med. Res. Rev. 2018; 38(2): 625–654. doi: 10.1002/med.21450 28586505

[18] Bonfils JP, Sauvaire Y, Baissac Y, Marner FJ. Phytochemistry. 1994; 37(3): 701–705. doi: 10.1016/s0031-9422(00)90342-x

[19] Wang J, Gao WY, Zhang J, Zuo BM, Zhang LM, Huang LQ. Advances in study of ginsenoside biosynthesis pathway in Panax ginseng C. A. Meyer. Acta Physiol. Plant. 2012; 34(2): 397–403. doi: 10.1007/s11738-011-0844-3

[20] Guo J, Zhang Q, Sun CZ, Wen J, Xie CX. Spatial variations of ginsenosides in *Panax ginseng* and their impact factors. Chinese Journal of Plant Ecology. 2017; 41(9): 995. CNKI:SUN:ZWSB.0.2017-09-007.

[21] Gao Y, Zhang T, Kang XP, Han M, Yang LM. Preliminary study on response and its mechanism of ginsenoside biosynthesis in Panax ginseng to water regulation. China Journal of Chinese Materia Medica. 2019; 44(13): 2768–2776. doi: 10.19540/j.cnki.cjcmm.20190323.103 31359689

[22] Wang EH, Li Q, Chi K, Yang H, Bi B, Wang HB, et al. Effect of low temperature stress on the cold resistance of drought. Ginseng research. 2016; 28(3): 2–4. doi: 10.19403/j.cnki.1671-1521.2016.03.001

[23] Lin H, Zhang Y, Han M, Yang LM. Aqueous ionic liquid based ultrasonic assisted extraction of eight ginsenosides from ginseng root. Ultrason Sonochem. 2013; 20(2): 680–684. doi: 10.1016/j.ultsonch.2012.10.003 23157924

[24] Kubo M, Tani T, Katsuki T, Keishi K, Shigeru I, Arichi. Histochemistry. I. Ginsenosides in ginseng (Panax ginseng CA Meyer, root). J. Nat. Prod. 1980; 43(2): 278–284. doi: 10.1021/np50008a006

[25] Song XW, Yao Y, Yu PC, Zhang W, Liu WF, Wang LY, et al. Sodium nitroprusside improved the quality of Radix Saposhnikoviae through constructed physiological response under ecological stress. Sci. Rep. 2023; 13(1): 15823. doi: 10.1038/s41598-023-43153-3 37740027 PMC10516912

[26] Li M, Zhao J, Tang Q, Zhang Q, Wang Y, Zhang J, et al. Lamivudine improves cognitive decline in SAMP8 mice: Integrating in vivo pharmacological evaluation and network pharmacology. J. Cell. Mol. Med. 2021; 25(17): 8490–8503. doi: 10.1111/jcmm.16811 34374199 PMC8419189

[27] Yu MH, Xue A, Zhao DP, Xu YM, Xue H, Jiang J, et al. Plasma metabolomics study of Buyang Huanwu Decoction improving learning and memory in D-gal model mice based on GC-MS. Journal of Hainan Medical University. 2022; 28(22): 1681–1687. doi: 10.13210/j.cnki.jhmu.20220617.001

[28] Zhang JJ, Chen KC, Zhou Y, Wei H, Qi MH, Wang Z, et al. Evaluating the effects of mitochondrial autophagy flux on ginsenoside Rg2 for delaying D-galactose induced brain aging in mice. Phytomedicine, 2022, 104: 154341. doi: 10.1016/j.phymed.2022.154341 35870376

[29] Sachdev S, Ansari SA, Ansari MI, Fujita M, Hasanuzzaman M. Abiotic stress and reactive oxygen species: Generation, signaling, and defense mechanisms. Antioxidants. 2021; 10(2): 277. doi: 10.3390/antiox10020277 33670123 PMC7916865

[30] Wang H, Chen Y, Hu W, Snider JL, Zhou Z. Short-term soil-waterlogging contributes to cotton cross tolerance to chronic elevated temperature by regulating ROS metabolism in the subtending leaf. Plant Physiol. Biochem. 2019; 139: 333–341. doi: 10.1016/j.plaphy.2019.03.038 30952085

[31] Ashraf MA. Waterlogging stress in plants: A review. Afr. J. Agril. Res. 2012; 7, 1976–1981. doi: 10.5897/AJARX11.084

[32] Anee TI, Nahar K, Rahman A, Mahmud JA, Bhuiyan TF, Alam MU, et al. Oxidative damage and antioxidant defense in Sesamum indicum after different waterlogging durations. Plants. 2019; 8(7): 196. doi: 10.3390/plants8070196 31261970 PMC6681296

[33] Miller G, Schlauch K, Tam R, Cortes D, Torres MA, Shulaev V, et al. The plant NADPH oxidase RBOHD mediates rapid systemic signaling in response to diverse stimuli. Sci. signaling. 2009; 2(84): ra45–ra45. doi: 10.1126/scisignal.2000448 19690331

[34] Li WY, Chen BX, Chen ZJ, Gao YT, Chen Z, Liu J. Reactive oxygen species generated by NADPH oxidases promote radicle protrusion and root elongation during rice seed germination. Int. J. Mol. Sci. 2017; 18(1): 110–127. doi: 10.3390/ijms18010110 28098759 PMC5297744

[35] Hu XY, Neill SJ, JY F, Cai WM, Tang ZC. The mediation of defense responses of ginseng cells to an elicitor from cell walls of Colletotricum lagerarium by plasma membrane NAD(P)H oxidases. Acta Botanica Sinica, 2003, 45(1): 32–39. doi: 10.1023/A:1022289509702

[36] Hu XY, Neill SJ, Cai WM, Tang ZC. Activation of plasma membrane NADPH oxidase and generation of H_2_O_2_ mediate the induction of PAL activity and saponin synthesis by endogenous elicitor in suspension-cultured cells of Panax ginseng. J Integr Plant Biol. 2003; 45(12): 1434. doi: 10.1071/SB03011

[37] Camejo D, Guzmán-Cedeño Á, Moreno A. Reactive oxygen species, essential molecules, during plant–pathogen interactions. Plant Physiol. Biochem. 2016; 103: 10–23. doi: 10.1016/j.plaphy.2016.02.035 26950921

[38] Li ZG, Yuan LX, Wang QL, Ding ZL, Dong CY. Combined action of antioxidant defense system and osmolytes in chilling shock-induced chilling tolerance in Jatropha curcas seedlings. Acta Physiol. Plant. 2013; 35: 2127–2136. doi: 10.1007/s11738-013-1249-2

[39] Xu WJ, Liu DD, Wang YN, Zhang CT, Tang J, Qiao HK, et al. Effects of different temperature treatments on flower bud differentiation and physiological property of *Physalis pubescens* L. Agricultural Research in the Arid Areas. 2023; 41(6): 88–96.

[40] Xue JY, Yang HN, Tang Y, Peng YJ, Wang LF. Effect of water stress on seed germination and physiological and biochemical characteristics of Leptochloa chinensis seeds. SEED. 2021; 40(5): 33–38. doi: 10.16590/j.cnki.1001-4705.2021.05.033

[41] Tewari RK, Lee SY, Hahn EJ, Paek KY. Temporal changes in the growth, saponin content and antioxidant defense in the adventitious roots of Panax ginseng subjected to nitric oxide elicitation. Plant Biotechnol Rep. 2007; 1: 227–235. doi: 10.1007/s11816-007-0036-1

[42] Szalai G, Kellős T, Galiba G, Kocsy G. Glutathione as an antioxidant and regulatory molecule in plants under abiotic stress conditions. J. Plant Growth Regul. 2009; 28: 66–80. doi: 10.1007/s00344-008-9075-2

[43] Ulrich K, Jakob U. The role of thiols in antioxidant systems. FREE RADICAL BIO MED. 2019; 140: 14–27. doi: 10.1016/j.freeradbiomed.2019.05.035 31201851 PMC7041647

[44] Asada K. The water-water cycle in chloroplasts: scavenging of active oxygens and dissipation of excess photons. Annu. Rev. Plant Biol. 1999; 50(1): 601–639. doi: 10.1146/annurev.arplant.50.1.601 15012221

[45] Jin YH, Tao DL, Hao ZQ, Ye J, Du Y, Liu H, et al. Environmental stresses and redox status of ascorbate. J Integr Plant Biol. 2003; 45(7): 795. doi: 10.1038/sj.onc.1206615

[46] Sinha AK, Jaggi M, Raghuram B, Tuteja N. Mitogen-activated protein kinase signaling in plants under abiotic stress. PLANT SIGNAL BEHAV. 2011; 6(2): 196–203. doi: 10.4161/psb.6.2.14701 21512321 PMC3121978

[47] Sairam R K, Dharmar K, Lekshmy S, Chinnusamy V. Expression of antioxidant defense genes in mung bean (Vigna radiata L.) roots under water-logging is associated with hypoxia tolerance. Acta Physiol. Plant. 2011; 33: 735–744. doi: 10.1007/s11738-010-0598-3

[48] Sparg S G, Light M E, Van Staden J. Biological activities and distribution of plant saponins. J Ethnopharmacol. 2004; 94(2–3): 219–243. doi: 10.1016/j.jep.2004.05.016 15325725

[49] Ma R, Fu B, Yang P, Fu BY, Zhao DQ, Yang PD, et al. Sucrose Induced HMGR to Promote Ginsenoside Biosynthesis in the Growth of Wild Cultivated Ginseng (Panax ginseng). J. Soil Sci. Plant Nutr. 2022; 22(2): 2255–2265. doi: 10.1007/s42729-022-00806-y

[50] Yang JL, Hu ZF, Zhang TT, Gu AD, Gong T, Zhu P. Progress on the studies of the key enzymes of ginsenoside biosynthesis. Molecules, 2018, 23(3): 589. doi: 10.3390/molecules23030589 29509695 PMC6017814

[51] Yin J, Zhang D, Zhuang J, Huang Y, Mu Y, Lv SW. Study on the correlation between gene expression and enzyme activity of seven key enzymes and ginsenoside content in ginseng in over time in Ji’an, China. Int. J. Mol. Sci. 2017; 18(12): 2682. doi: 10.3390/ijms18122682 29232922 PMC5751284

[52] Yang LL, Zhang T, Yang LM, Han M. Effects of ecological factors on ginsenosides synthesis and its key enzyme genes expression. Chinese Traditional and Herbal Drugs, 2017: 4296–4305. doi: 10.7501/j.issn.0253-2670.2017.20.026

[53] Kotake T, Yamanashi Y, Imaizumi C, Tsumuraya Y. Metabolism of L-arabinose in plants. J Plant Res. 2016; 129: 781–792. doi: 10.1007/s10265-016-0834-z 27220955 PMC5897480

[54] Hsieh YSY, Zhang Q, Yap K, Shirley NJ, Lahnstein J, Nelson CJ, et al. Genetics, transcriptional profiles, and catalytic properties of the UDP-arabinose mutase family from barley. Biochemistry. 2016; 55(2): 322–334. doi: 10.1021/acs.biochem.5b01055 26645466

[55] Zhao J, Davis LC, Verpoorte R. Elicitor signal transduction leading to production of plant secondary metabolites. Biotechnol. Adv. 2005; 23(4): 283–333. doi: 10.1016/j.biotechadv.2005.01.003 15848039

[56] Chang J W, Lee G, Coukos JS, Moellering RE. Profiling reactive metabolites via chemical trapping and targeted mass spectrometry. Anal Chem. 2016; 88(13): 6658–6661. doi: 10.1021/acs.analchem.6b02009 27314642 PMC4998964

[57] O’Leary B, Fedosejevs ET, Hill AT, Bettridge J, Park J, Rao SK, et al. Tissue-specific expression and post-translational modifications of plant- and bacterial-type phosphoenolpyruvate carboxylase isozymes of the castor oil plant, *Ricinus communis* L. J. Exp. Bot. 2011; 62(15):5485–5495. doi: 10.1093/jxb/err225 21841182 PMC3223045

[58] Li Y, Zhang D, Li L, Han YL, Dong XN, Yang L, et al. Ginsenoside Rg1 ameliorates aging‑induced liver fibrosis by inhibiting the NOX4/NLRP3 inflammasome in SAMP8 mice. Mol. Med. Rep. 2021; 24(5): 1–14. doi: 10.3892/mmr.2021.12441 34523690 PMC8456316

[59] Zhu J, Mu X, Zeng J, Xu CY, Liu J, Zhang MS, et al. Ginsenoside Rg1 prevents cognitive impairment and hippocampus senescence in a rat model of D-galactose-induced aging. PloS one. 2014; 9(6): e101291. doi: 10.1371/journal.pone.0101291 24979747 PMC4076296

[60] Gong Y S, Guo J, Hu K, Gao YQ, Xie BJ, Sun ZD, et al. Ameliorative effect of lotus seedpod proanthocyanidins on cognitive impairment and brain aging induced by D-galactose. Exp. Gerontol. 2016, 74: 21–28. doi: 10.1016/j.exger.2015.11.020 26657492

[61] Hao MQ, Ding CB, Peng XJ, Chen HY, Dong L, Zhang Y, et al. Ginseng under forest exerts stronger anti-aging effects compared to garden ginseng probably via regulating PI3K/AKT/mTOR pathway, SIRT1/NF-κB pathway and intestinal flora. Phytomedicine, 2022, 105: 154365. doi: 10.1016/j.phymed.2022.154365 35930860

